# Affinity‐Based Protein Profiling Reveals IDH2 as a Mitochondrial Target of Cannabinol in Receptor‐Independent Neuroprotection

**DOI:** 10.1002/chem.202501143

**Published:** 2025-05-19

**Authors:** Eva Schaller, Stephanie Lamer, Andreas Schlosser, Christian Stigloher, Pamela Maher, Michael Decker

**Affiliations:** ^1^ Julius‐Maximilians‐Universität Würzburg (JMU) Institute for Pharmacy and Food Chemistry Pharmaceutical and Medicinal Chemistry Am Hubland 97074 Würzburg Germany; ^2^ Julius‐Maximilians‐Universität Würzburg (JMU) Rudolf‐Virchow‐Zentrum – Center for Integrative and Translational Bioimaging 97080 Würzburg Germany; ^3^ Julius‐Maximilians‐Universität Würzburg (JMU) Biocenter/Theodor‐Boveri‐Institute Imaging Core Facility Am Hubland 97074 Würzburg Germany; ^4^ Cellular Neurobiology Laboratory The Salk Institute for Biological Studies 10010 N Torrey Pines Road La Jolla California 92037 USA

**Keywords:** alzheimer's disease, mitochondria, natural products, neuroprotection, proteomics

## Abstract

Phytocannabinoids are attracting growing attention because of their potential for treatment of neurodegenerative diseases. Among them, the “minor” cannabinoid, cannabinol (CBN), has emerged as a promising neuroprotective agent, acting independently of classical cannabinoid receptors through as‐yet unidentified mitochondrial targets. To uncover the molecular basis of its neuroprotective effects, we designed and synthesized a chemical probe based on CBN, incorporating a minimalist diazirine linker. Functional assays confirmed that the probe retains CBN's mitochondrial activity and exhibits strong mitochondrial enrichment, as demonstrated by fluorescence microscopy and click‐correlative light and electron microscopy (click‐CLEM). By affinity‐based protein profiling (A*f*BPP), we identified isocitrate dehydrogenase 2 (IDH2) as a key mitochondrial target of CBN. This finding was further substantiated by siRNA knockdown studies, which revealed that the absence of IDH2 partially phenocopies CBN's effects, validating its role as a critical mediator of CBN's neuroprotective activity.

## Graphical abstract

A chemical probe CBN‐P based on the neuroprotective cannabinoid cannabinol (CBN) was developed and synthesized. CNB‐P was found to have a similar mechanism of action as the parent compound and was used in click‐CLEM microscopy studies and affinity‐based protein profiling to identify the mitochondrial protein isocitrate dehydrogenase 2 (IDH2) as one target of CBN.

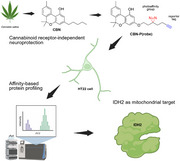

## 1 Introduction

Phytocannabinoids have gained tremendous therapeutic interest lately due to their various effects on human biology.^[^ ^1^ ^]^ They show anti‐inflammatory, anxiolytic, neuroprotective, and potential antimicrobial effects.^[^ ^1^, ^2^ ^]^ Many of these effects are mediated via the canonical cannabinoid receptors 1 and 2 (CB_1_R, CB_2_R). However, other targets of phytocannabinoids, including tetrahydrocannabinol (THC) and cannabidiol (CBD), have been found, that are not CBRs. CBD, for example, acts on various receptors as well as enzymes, transporters, and ion channels.^[^ ^3^ ^]^ Furthermore, the CBD‐derivative CIAC001, which showed improved anti‐neuroinflammatory properties, was found to act on pyruvate kinase M2 (PKM2) as analyzed by affinity‐based protein profiling (A*f*BPP).^[^ ^4^ ^]^ Both phytocannabinoids, THC and CBD, show great potential in the treatment of Alzheimer's disease (AD).^[^ ^5^ ^]^ But especially since THC shows strong activity on CB_1_R and CB_2_R, its potential clinical application is limited due to its psychoactive side effects. Therefore, the research focus has shifted more to nonpsychoactive cannabinoids like cannabinol (CBN) which occurs in minor amounts in the plant *Cannabis sativa*. CBN, as the oxidized form of THC, shows neuroprotective effects in vitro as demonstrated in phenotypic screening assays.^[^ ^6^ ^]^ In that study, several phytocannabinoids were evaluated regarding their potential neuroprotective effects.^[^ ^6^ ^]^ CBN as a nonpsychoactive compound was further evaluated and the mode of action underlying CBN‐mediated neuroprotection was thoroughly investigated.^[^ ^7^ ^]^ In several assays, the effect of CBN on the mouse hippocampal cell line HT22 with and without the ferroptosis‐inducing compound RSL3 was examined resulting in the finding that the neuroprotective effect *in vitro* is mainly exerted through the action of CBN on mitochondria. Moreover, CBN requires functional mitochondria to protect the cells against the RSL3 insult. Furthermore, it was demonstrated that the neuroprotection was not CBR‐mediated, because HT22 cells lack CBRs^[^ ^7^ ^]^ further indicating that phytocannabinoids can exert positive effects through non‐CBR‐targets. Apart from CBRs, CBN is known to act as an agonist of TRPA1 and as an antagonist of TPRM8 channels.^[^ ^8^ ^]^ Furthermore, it acts on peroxisome proliferator‐activated receptors (PPARs) and serotonin receptors.^[^ ^9^ ^]^ But the target(s) relevant for the neuroprotective effects of CBN still remain elusive. Therefore, we chose to apply A*f*BPP using photoaffinity labeling for target identification, since CBN cannot bind covalently to its target thereby impeding the use of activity‐based protein profiling.^[^ ^10^ ^]^ We chose the diazirine functional group as the photoreactive group due to its small size and comparably long wavelengths needed for activation.^[^ ^11^ ^]^ In this work, a photoaffinity probe based on the cannabinoid CBN (CBN‐P) was synthesized using a minimalist photoaffinity labeling (PAL) linker containing a diazirine group and an alkyne moiety for subsequent modification^[^ ^12^ ^]^ and its mode of action was compared to the parent molecule CBN. Additional fluorescence microscopy and click‐correlative light and electron microscopy (click‐CLEM) studies^[^ ^13^ ^]^ showed strong enrichment of CBN‐P in the mitochondria encouraging us to search for a mitochondrial target in LC‐MS/MS data of the *Af*BPP. Isocitrate dehydrogenase 2 (IDH2) was identified as a mitochondrial, non‐CBR target of CBN and verified by siRNA knock‐down studies.

## 2 Results and Discussion

### 2.1 Design and Synthesis

**Scheme 1:**
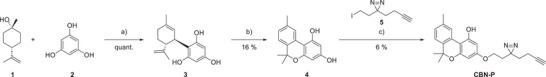
Synthesis of CBN‐P. (A) Reagents and conditions: 1. BF_3_ OEt_2_, THF, −10 °C, 3 hours; (B) I_2_, toluene, 130 °C, overnight; (C) K_2_CO_3_, minimalist linker **5**, acetone, 50 °C, overnight.

Aiming to keep the structure of CBN‐P as close as possible to CBN itself, the five‐carbon aliphatic chain of CBN was exchanged for a minimalistic PAL linker **5**.^[^ ^12^ ^]^ Other potential probe structures and unsuccessful synthesis routes can be found in the Supporting Information, Schemes S1–S6. The linker **5** was synthesized following literature procedures^[^ ^12^, ^14^ ^]^ with modification in the diazirine formation step to omit using liquid ammonia (Supporting Information, Scheme S7). Instead, 7N ammonia in methanol and *t*BuOCl as oxidant was used according to *Ibert* et al.^[^ ^15^ ^]^ The CBN‐core was synthesized from phloroglucinol and *trans*‐β‐terpineol followed by a low‐yielding oxidation with iodine.^[^ ^16^, ^17^ ^]^ Different oxidants like DDQ, *o*‐chloranil and other benzoquinone‐based oxidants were tested but did not yield the desired product. The linker was connected in the last step via a Williamson ether synthesis (Scheme 1). The correct substitution pattern was verified with NOESY NMR spectroscopy (Supporting Information, Figure S1). Low yields in the last step can be explained by lack of regioselectivity and degradation of the diazirine group under the reaction conditions applied. Unfortunately, milder reaction conditions did not lead to any product formation at all, so these conditions were determined as the sweet spot between no product and diazirine‐degradation.

### 2.2 Cell‐Based Assays to Confirm a Similar Mode of Action

**Figure 1:**
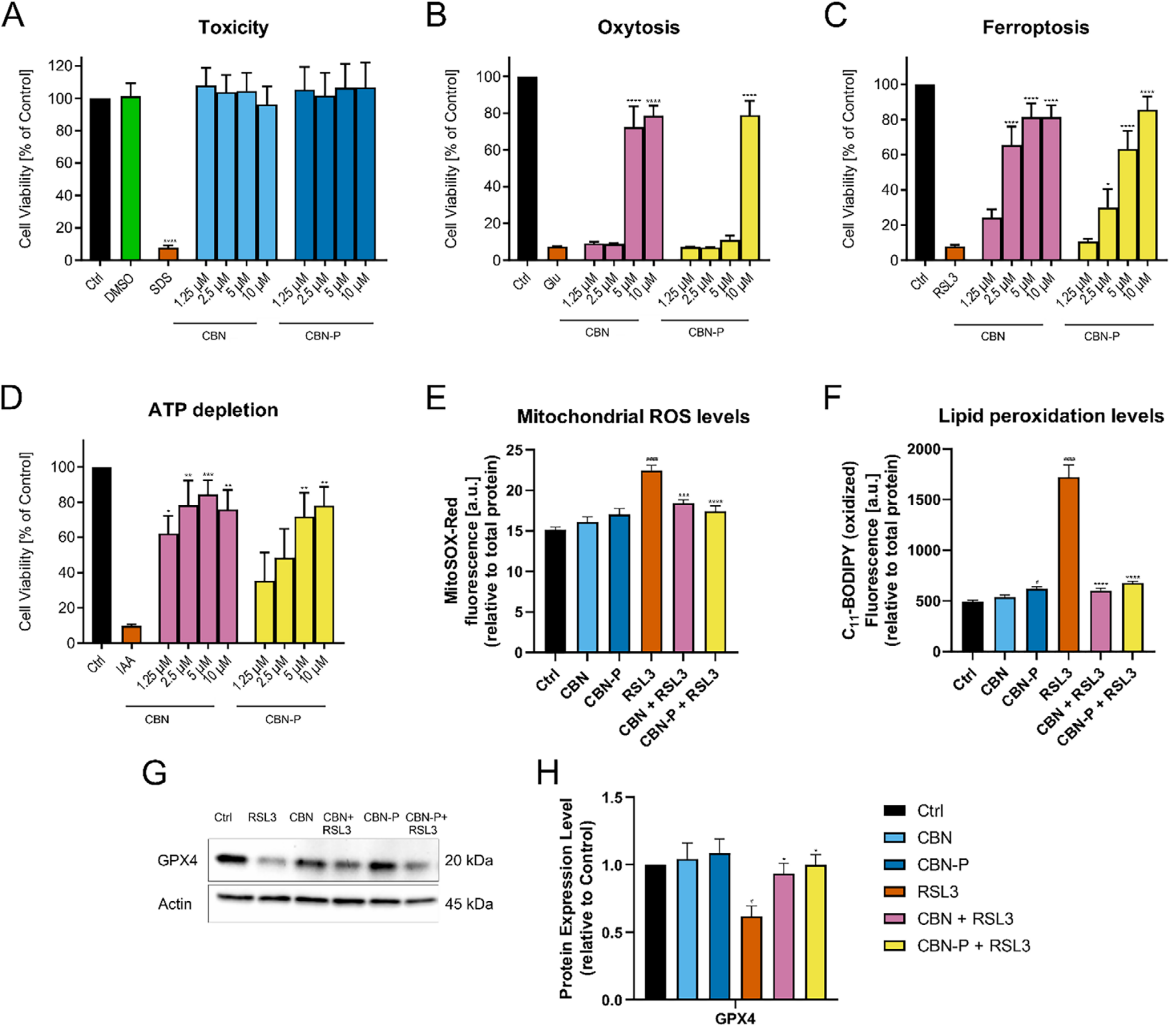
(A–D) Phenotypic screening assays in HT22 hippocampal neuronal cells. (A) Neurotoxicity of CBN and CBN‐P. (B–D) Neuroprotective effects of CBN and CBN‐P against. (B) Oxytosis induced by 5 mM glutamate, (C) ferroptosis induced by 100 nM RSL3, and (D) *in vitro*‐ischemia induced by 17.5 µM iodoacetic acid. Data are presented as means ±SEM of three independent experiments. Statistical analysis was performed using one‐way ANOVA followed by Dunnett's multiple comparison post‐test using GraphPad Prism 10 referring to untreated control cells, (A) or cells treated with the respective insult only and (B,C,D) (orange). Levels of significance: *p < 0.05; **p < 0.01; ***p < 0.001, ****p < 0.0001. (E) Mitochondrial ROS levels upon different treatments in the cells for 16 hours. Data were normalized to total protein/well and are the mean of 16 replicates per condition ± SEM. (F) Cellular lipid peroxidation levels upon different treatment conditions of the cells for 16 hours. Data were normalized to total protein/well and are the mean of 16 replicates per condition ±SEM. (G) Western blot data of GPX4 and actin (*n* = 4). Protein levels were measured upon different treatment conditions of the cells for 16 hours. (H) Densitometric quantification of the Western blots. Data were normalized to actin and are the mean ±SEM. Data for E, F, H were analyzed by one‐way ANOVA with Tukey's multiple comparison test. *#p < 0.05, ####p < 0.0001* relative to vehicle control; *p < 0.05; **p < 0.01; ***p < 0.001, ****p < 0.0001 relative to the RSL3 treatment.

For using a chemical probe to identify actual targets of the parent molecule it is essential to keep the mechanism of action the same. This was evaluated by using the same phenotypic screening assays that were used to identify the neuroprotective effect of CBN originally.^[^ ^6^, ^18^ ^]^ First, CBN‐P was tested for neurotoxicity on HT22 cells which lack CBRs showing no toxicity up to 10 µM overnight (Figure 1A) and up to 60 µM for 2 h (Supporting Information, Figure S2). Neuroprotection against oxytosis/ferroptosis and ATP depletion was also examined. Oxytosis induced by glutamate and ferroptosis induced by RSL3 are forms of oxidative stress‐induced cell death that mimic the increased oxidative stress in the ageing brain that is exacerbated in AD.^[^ ^19^, ^20^ ^]^ CBN‐P is active against both stressors–glutamate and RSL3–but at slightly higher concentrations than CBN itself (Figure 1B,C). Applying iodoacetic acid to HT22 cells leads to ATP depletion similar to the reduced energy availability of the ageing brain, a major risk factor for AD.^[^ ^21^ ^]^ In this assay as well, CBN‐P showed a slightly decreased but still considerable neuroprotective activity (Figure 1D).

For further evaluation of the probe's effects on oxidative stress, levels of mitochondrial reactive oxygen species (ROS) and lipid peroxidation–both markers of increased oxidative stress induced by RSL3–were examined: CBN and CBN‐P had no effect on baseline ROS and lipid peroxidation levels but were able to prevent the increases in ROS levels induced by RSL3 (Figure 1E,F). Furthermore, the effect of CBN and CBN‐P on the expression of the antioxidant protein GPX4, relevant to the ferroptosis pathway, was investigated. No effect on the baseline levels was observed, but prevention of the reduction in GPX4 expression induced by RSL3 (Figure 1G) was seen with both CBN and CBN‐P. Therefore, the effects of CBN and CBN‐P on oxidative stress in HT22 cells are comparable, supporting the use of CBN‐P as a chemical probe for CBN.

Because neuroprotective effects of CBN appear to be mediated through its effects on mitochondria,^[^ ^7^ ^]^ several mitochondrial properties were analyzed to demonstrate a similar activity of CBN‐P. First, we looked at mitochondrial calcium homeostasis which is disrupted in the ageing brain and especially in Alzheimer's disease (AD).^[^ ^22^ ^]^ Directly analyzing mitochondrial calcium levels showed that CBN‐P –like CBN itself– showed no effect on baseline calcium levels. The increased calcium levels after treatment with RSL3 were prevented by addition of CBN or CBN‐P, respectively (Supporting Information, Figure S3). The mitochondrial calcium uniporter (MCU) as a key calcium channel is strongly relevant for intra‐mitochondrial calcium levels and its expression is increased in AD.^[^ ^23^ ^]^ Therefore, we analyzed its expression levels under different conditions showing no effect of CBN and CBN‐P by themselves on expression levels, but an ability of both of these compounds to prevent the increased expression induced by RSL3 (Supporting Information, Figure S3).

Assessing the number and biogenesis of mitochondria, we looked at the expression of the mitochondrial markers TOM20 and SIRT1. The latter is strongly involved in the formation of new mitochondria via the AMPK/SIRT1/PGC‐1α pathway.^[^ ^24^ ^]^ CBN‐P showed a comparable effect on these proteins to CBN with and without the treatment with RSL3 (Supporting Information, Figure S4).

As a dynamic network, mitochondria constantly undergo fusion and fission processes in cells that play an important role in maintaining healthy mitochondria.^[^ ^25^ ^]^ These processes are impaired in AD, leading to a deterioration in the function of mitochondria and therefore less energy availability in the brain.^[^ ^26^, ^27^ ^]^ Analyzing the expression of fusion (OPA1, MFN2) and fission (DRP1, MFF) proteins with and without treatment with RSL3 shows that both, CBN and CBN‐P, slightly increased baseline expression of these proteins. CBN and CBN‐P furthermore prevented the reduction of expression mediated by RSL3 (Supporting Information, Figure S5).

**Figure 2:**
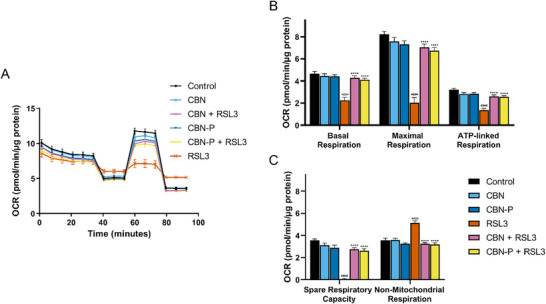
(A) Mitochondrial oxygen consumption rate (OCR) profiles in HT22 cells after different treatments for 16 hours. Data were normalized to total protein/well and are the mean of 16 replicates per condition ±SEM. (B, C) Graphs for basal respiration, maximal respiration, ATP‐linked respiration, spare respiratory capacity, and nonmitochondrial respiration in HT22 cells. Data for B,C were analyzed by one‐way ANOVA with Tukey's multiple comparison test *####p < 0.0001* relative to vehicle control; ****p < 0.0001 relative to the RSL3 treatment.

Mitochondrial ATP production via oxidative phosphorylation (OXPHOS) (mitochondrial bioenergetics) is important for energy production in cells. Disruption of bioenergetics can therefore play a role in cell damage and death.^[^ ^28^ ^]^ Assessing the effect of CBN and CBN‐P on mitochondrial bioenergetics, a seahorse mitochondrial stress test was performed. Sequential treatment of the cells with OXPHOS inhibitors (*i.e*., oligomycin, FCCP, rotenone, and antimycin A) and analysis of the oxygen consumption rate normalized to total protein allows investigation of basal, maximal, ATP‐linked, and nonmitochondrial respiration, as well as spare respiratory capacity. CBN and CBN‐P slightly, but not significantly, decreased maximal respiration and spare respiratory capacity, whereas treatment with RSL3 strongly diminished basal, maximal, and ATP‐linked respiration and spare respiratory capacity, but increased non‐mitochondrial respiration. Applying CBN or CBN‐P together with RSL3 counteracted these effects (Figure 2).

These results of phenotypic screening and mitochondrial assays strongly suggest that CBN‐P acts with a very similar mode of action as CBN underlining the compounds’ effects in neuroprotection. Furthermore, the various assays showed an effect of CBN‐P on mitochondrial Ca^2+^ homeostasis, biogenesis, fusion and fission, and bioenergetics, respectively, implying direct effects of CBN‐P on mitochondria.

### 2.3 Fluorescence Microscopy and Click‐CLEM

**Figure 3:**
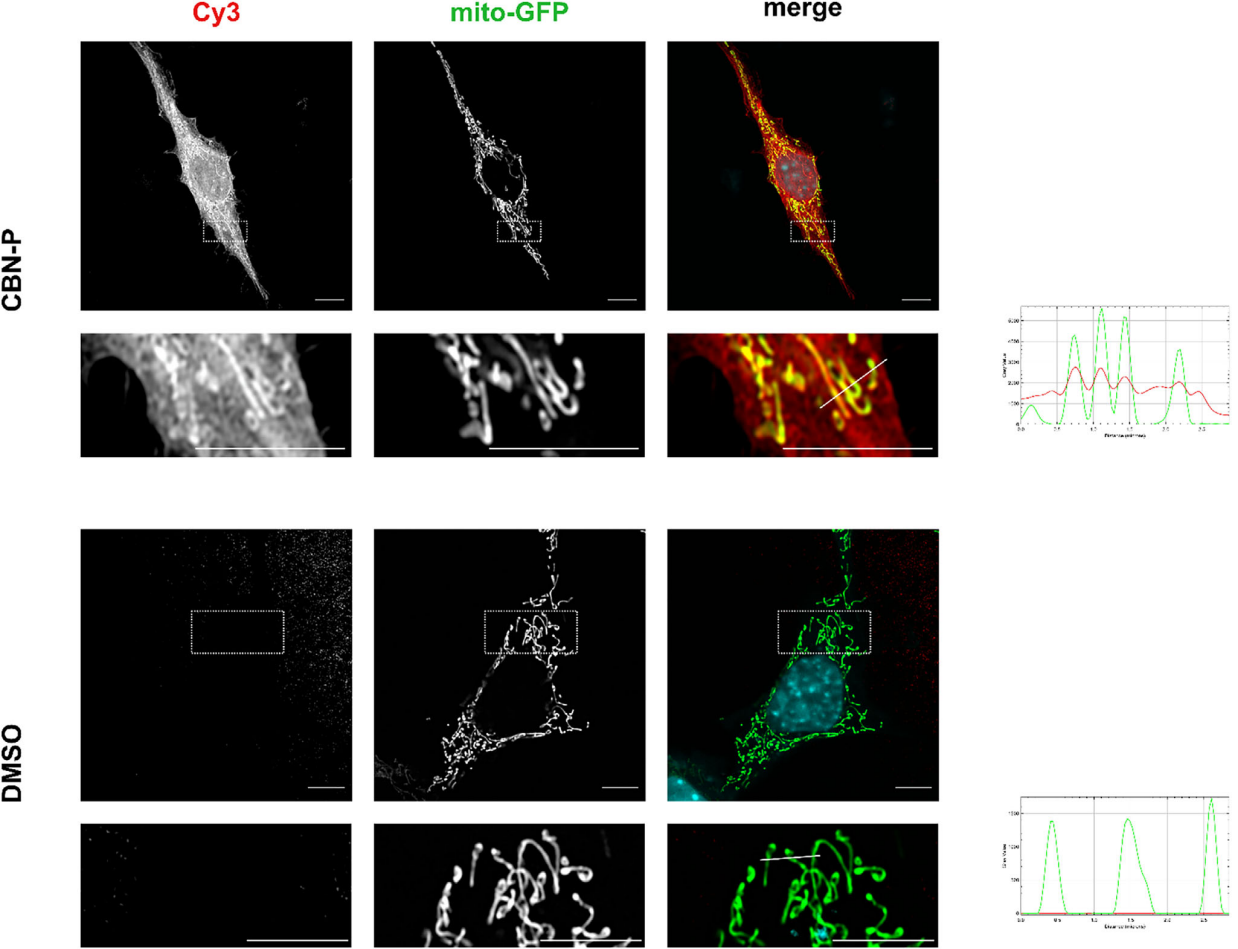
Microscopic analysis of HT22‐*mito*‐GFP cells with CBN‐P. Representative microscopic images of HT22‐*mito*‐GFP cells treated with 5 µM of CBN‐P for 30 minutes. Red signals derive from Cy3 attached to the probes representing protein adducts; green signals correspond to mitochondria‐targeted GFP. Mitochondrial structures are clearly labelled in cells incubated with CBN‐P compared to the absence of a signal in DMSO‐treated cells. For analysis of colocalization, a line was plotted in the merged image and fluorescence intensity quantified along this line displayed in the chart on the right. Scale bar equals 5 µm.

To get an initial idea about the intracellular localization of CBN‐P, we conducted fluorescence microscopy studies on HT22 cells stably expressing GFP in mitochondria (*mito*‐GFP). Therefore, *mito*‐GFP HT22 cells were treated with 5 µM CBN‐P and underwent a copper‐catalyzed azido‐alkyne cycloaddition (CuAAC) with Cy3‐azide after crosslinking of the probe. Results are displayed in Figure 3 and show a clear enrichment of CBN‐P in the mitochondrial‐rich areas of the cells compared to DMSO‐treated control cells.

**Figure 4:**
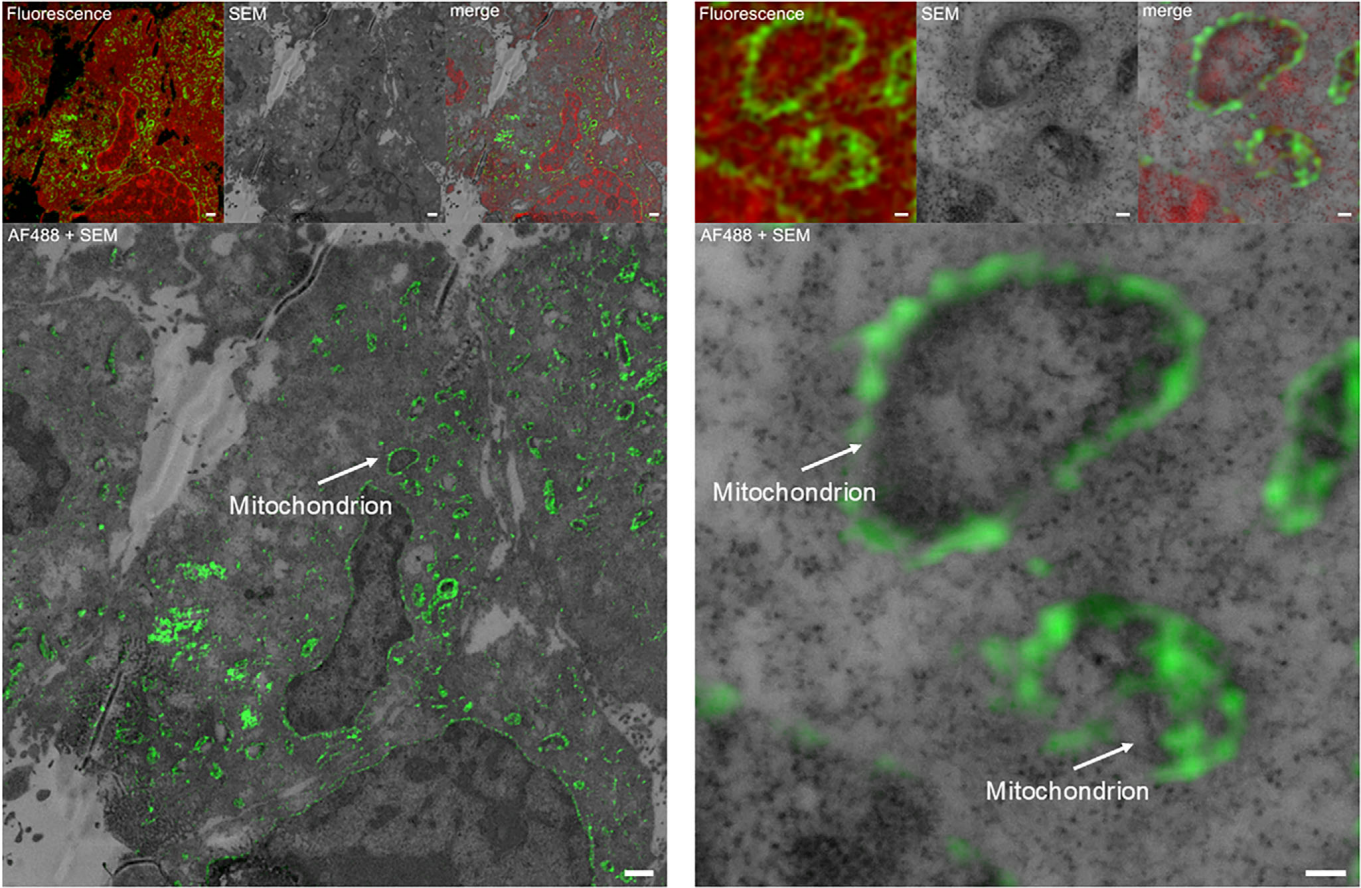
Click‐correlative light and electron microscopy images of HT22 cells treated with 5 µM of CBN‐P.^[^ ^13^ ^]^ Green signal: AF488 attached to the probe; red signal: methyl green (DNA). Upper row shows the image of the merged fluorescence channels after channel alignment taken with SIM, the SEM image, and the merged image showing both fluorescence channels correlated with the SEM image after unbiased correlation; AF488 +SEM only displays the probe signal and the SEM image. Images on the right are higher magnified sections of images on the left. Scale bar equals 1 µm (left) or 100 nm (right).

These results were further supported by a click‐CLEM experiment according to our previously developed protocol enabling visualization of small molecule probes by a combined approach using fluorescence and electron microscopy.^[^ ^13^ ^]^ Signal overlap of probe‐derived fluorescence signal with the cellular ultrastructure imaged with electron microscopy gives a more unbiased picture of co‐localization than fluorescence microscopy alone. In the correlated images of fluorescence and electron microscopy images (Figure 4), enrichment of CBN‐P at the mitochondria becomes apparent (DMSO control can be found in the Supporting Information, Figure S6).

These imaging studies indicate that CBN not only directly acts on mitochondria but is also localized there after addition to cells.

### 2.4 Affinity‐Based Protein Profiling Revealing IDH2 as a Mitochondrial Target

Before conducting A*f*BPP studies, we further validated the use of CBN‐P as a suitable probe for CBN by carrying out a CuAAC with Cy3‐azide on HT22 cell lysates. Protein adducts were visualized with Western blot analysis using fluorescence imaging. Therefore, HT22 cells were treated with increasing concentrations of CBN‐P (5, 10, 20, 40, and 80 µM) for 4 hours, crosslinked using 365 nm light, lyzed, and submitted to CuAAC with 20 µM Cy3‐azide. As expected, we could see a range of protein interactions with CBN‐P, some of which could be prevented by preincubation with a higher CBN concentration. This implies that CBN‐P does indeed interact with the same proteins as CBN. We further repeated the assay with 10 µM of CBN‐P but tested different incubation times (1, 2, and 4 hours) and decided to use 1 hour incubation time for the A*f*BPP assay by which time most protein interactions had occurred (Supporting Information, Figure S7).

After demonstrating that CBN‐P shows a similar mode of action to CBN and appears to interact with the same proteins, we conducted target identification following an A*f*BPP work scheme analogous to Gunesch et al.^[^ ^29^ ^]^ with an additional crosslinking step followed by an affinity pulldown. For this experiment, HT22 cells were incubated with 60 µM of CBN‐P or a comparable DMSO‐concentration as control for 1 hour, respectively. For a displacement assay, cells were first incubated with 120 µM of CBN for 30 min before treatment with 60 µM CBN‐P. After incubation, the probe was crosslinked with 365 nm light, then cells were lyzed and subjected to CuAAC with biotin‐azide. Bound proteins were purified on streptavidin magnetic beads and eluted. Samples were further processed in a single‐pot, solid‐phase‐enhanced sample‐preparation^[^ ^30^ ^]^ before analysis by nanoLC‐MS/MS. In total, 641 proteins were found. Of these, 14 were significantly enriched in both replicates over both controls (Supporting Information, Figure S8). Searching for a mitochondrial target, we applied the mouse MitoCarta3.0 database^[^ ^31^ ^]^ as a filter and then chose IDH2 as a target protein for further evaluation because it was significantly enriched over both controls (DMSO and displacement‐assay with CBN).

### 2.5 Target Evaluation of IDH2

IDH2 is a protein involved in the tricarboxylic acid cycle (TCA) and catalyzes the oxidative decarboxylation of isocitrate to α‐ketoglutarate (α‐KG).^[^ ^32^ ^]^ Normally, IDH2 can catalyze two distinct reactions: the oxidative decarboxylation and the reverse reaction, that is, the reductive carboxylation (RC) of α‐KG to isocitrate. The RC pathway is particularly prominent in cells with mitochondrial defects and is associated with increased oxidative stress and lipogenesis.^[^ ^33^, ^34^ ^]^ Inhibition of fatty acid synthesis is known to be protective against the oxytosis/ferroptosis pathway.^[^ ^35^ ^]^ Furthermore, inhibition of RC by mitochondrial uncouplers also protects against oxytosis/ferroptosis.^[^ ^36^ ^]^

Mutations of IDH2 are implicated in the development of various cancer types. Instead of catalyzing the formation of α‐KG, mutant IDH2 generates β‐hydroxyglutarate, leading to hypermethylation of target proteins and impaired cellular differentiation.^[^ ^32^ ^]^ Conversely, downregulation of IDH2 also seems to decrease the pro‐inflammatory response in BV2 and primary microglia cells.^[^ ^37^ ^]^

A study in *Drosophilia* has demonstrated that IDH2 knockdown impairs the formation of the OXPHOS complex, resulting in increased ROS accumulation and the induction of the ferroptosis pathway.^[^ ^38^ ^]^

**Figure 5:**
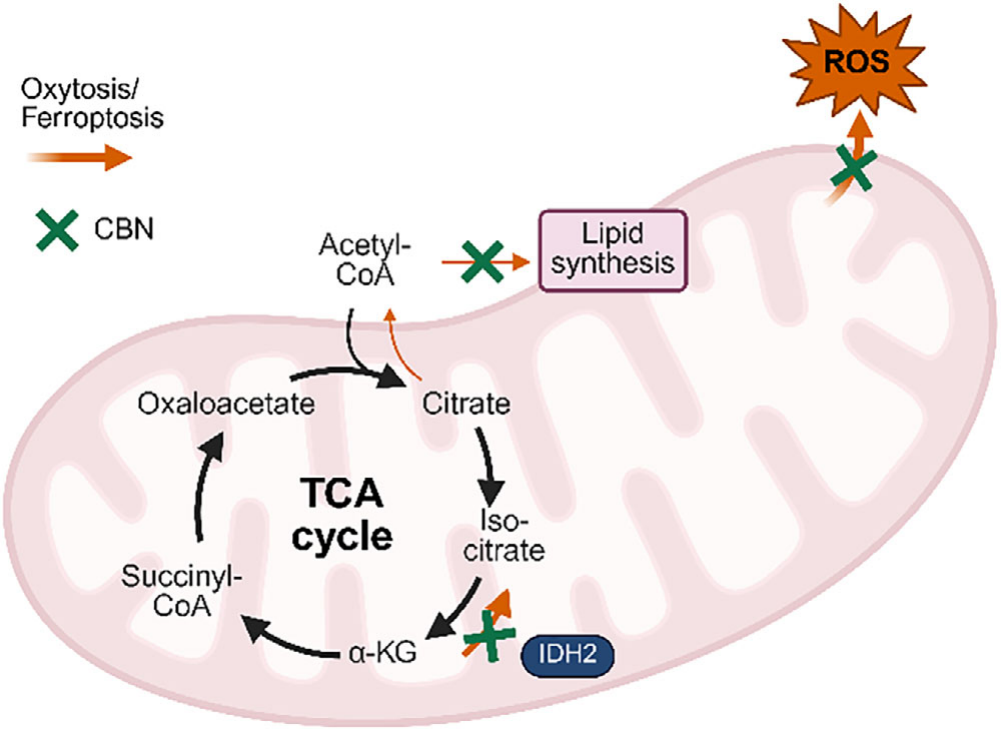
Proposed mechanism of neuroprotection via IDH2. Schematic representation of the TCA cycle in mitochondria including the oxidative decarboxylation of isocitrate to α‐ketoglutarate by the enzyme IDH2. Oxytosis/ferroptosis conditions could cause IDH2 to catalyze the reverse reductive carboxylation reaction from α‐ketoglutarate to iso‐citrate (orange arrows) followed by increased lipogenesis and ROS production. Inhibition of IDH2 by CBN (green cross) may counteract these effects thereby protecting cells from oxytosis/ferroptosis.

Given these seemingly contradictory observations, it was hypothesized that under oxytosis/ferroptosis conditions, IDH2 activity is more skewed to the RC pathway. Therefore, inhibiting IDH2 could potentially offer protection against oxytosis/ferroptosis (a proposed mechanism is illustrated in Figure 5).

**Figure 6:**
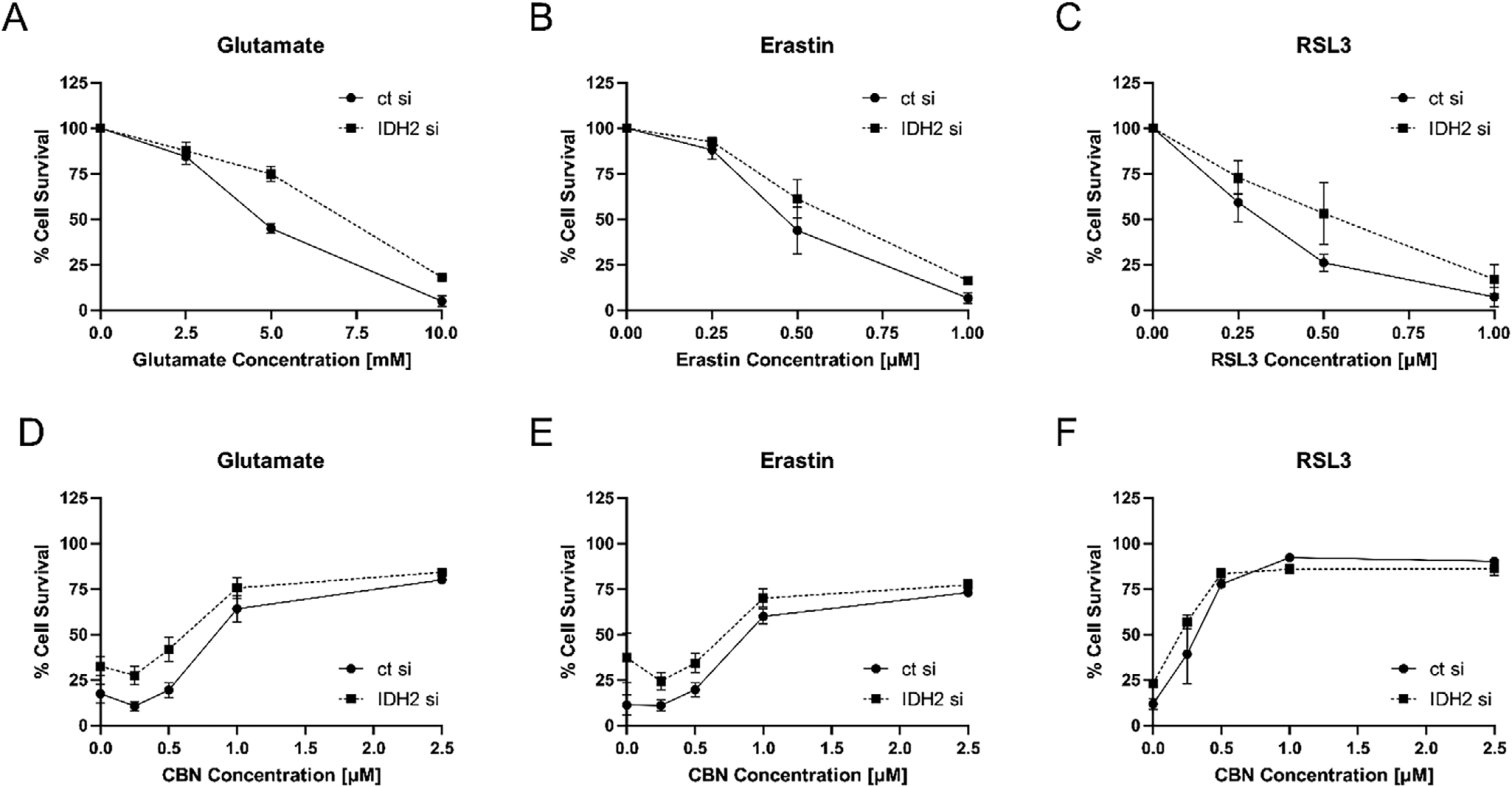
Transfection of HT22 cells with IDH2 siRNA and investigations into neuroprotection. (A) IDH2 knockdown leads to protection against glutamate as an inducer of oxytosis, (B) erastin, and (C) RSL3 as inducers of ferroptosis. IDH2 knockdown and control cells were treated with increasing concentrations of CBN in the presence of (D) 10 mM glutamate, (E) 1 µM erastin, or (F) 1 µM RSL3. Data are presented as means ±SD of three independent experiments.

HT22 cells transfected with IDH2 siRNA were tested for effects on oxytosis/ferroptosis. Efficacy of the knockdown can be seen in Supporting Information, Figure S9. Knocking down IDH2 protected against glutamate, erastin, and RSL3 as inducers of the oxytosis/ferroptosis pathway (Figure 6A–C). Significantly more cells survived in the IDH2 siRNA treated cells compared to cells treated with control siRNA. These findings support both the idea that IDH2 inhibition is protective against oxytosis/ferroptosis and that it is involved in the protective effects of CBN.

We then treated IDH2 knockdown cells with CBN in the presence of the different inducers of oxytosis/ferroptosis. The data showed very similar behavior for IDH knockdown cells and control cells when treated with increasing CBN concentrations (Figure 6D–F). This further supports our hypothesis that IDH2 is indeed a target of CBN because knockdown and inhibition of IDH2 by CBN showed almost identical protection against oxytosis/ferroptosis insults. However, additional targets of CBN might well be involved in neuroprotection since cell survival of IDH2 knockdown cells increases with increased CBN concentration. This does not come as a surprise because many natural products exhibit their effects through multiple targets.^[^ ^29^, ^39^ ^]^

## 3 Conclusion

In conclusion, we have successfully designed and synthesized a chemical probe, CBN‐P, based on the neuroprotective cannabinoid CBN, incorporating a minimalist diazirine‐alkyne linker for CuAAC reactions. Phenotypic screening assays mimicking key aspects of ageing and neurodegeneration revealed that CBN‐P closely mirrors the parent compound's activity. By employing assays focused on mitochondrial functions, including calcium homeostasis, biogenesis, bioenergetics, and fusion/fission, we further confirmed that introduction of the minimalist linker doesnot impede the probe's mechanism of action.

Advanced imaging techniques, such as fluorescence microscopy on *mito*‐GFP cells and click‐CLEM, demonstrated robust mitochondrial enrichment of CBN‐P, while LC‐MS/MS analysis post‐A*f*BPP revealed 14 potential target candidates. Among these, IDH2 emerged as a mitochondrial target. Knock‐down studies underscored IDH2's pivotal role in cell survival and its absence partially phenocopied the effects of CBN in HT22 cells. Through A*f*BPP, we pinpointed IDH2 as a key player in the neuroprotective effects of CBN, shedding light on its involvement in the TCA cycle and mitochondrial integrity.

These findings mark a significant step forward in unraveling the cellular mechanisms and interaction partners of phytocannabinoids beyond the classical CBR pathways. Identifying IDH2 as a target of CBN paves the way for future investigations into its role–and that of other TCA‐associated enzymes–in neurodegenerative diseases such as AD. Furthermore, click‐CLEM microscopy and this A*f*BPP approach offer a powerful platform for uncovering targets of other nonpsychoactive, neuroprotective cannabinoids, enabling deeper exploration of their therapeutic potential.

## Supporting Information

The authors have cited additional references within the Supporting Information.^[^ ^40^, ^41^, ^42^, ^43^, ^44^, ^45^, ^46^, ^47^ ^]^

## Conflict of Interests

The authors declare no conflict of interest.

## Supporting information



Supporting Information

## Data Availability

The data that support the findings of this study are available in the supporting information of this article.
