# Supplementary material for: Affinity‐Based Protein Profiling Reveals IDH2 as a Mitochondrial Target of Cannabinol in Receptor‐Independent Neuroprotection
Source: Chemistry. 2025 May 19;31(33):e202501143. doi: 10.1002/chem.202501143 (PMC12160979; doi:10.1002/chem.202501143)
Supplement: Supplementary file 1 — Supporting Information [file CHEM-31-e202501143-s001.docx]

[Unsuccessful CBN-Probe Synthesis Routes 2](#_Toc195774577)

[Experimental Procedures 5](#_Toc195774578)

[Figure S1: NOESY of CBN-P 18](#_Toc195774579)

[Figure S2: Toxicity of CBN-P on HT22 cells 19](#_Toc195774580)

[Figure S3: Mitochondrial Calcium Homeostasis 19](#_Toc195774581)

[Figure S4: Mitochondrial Biogenesis 20](#_Toc195774582)

[Figure S5: Mitochondrial Dynamics 20](#_Toc195774583)

[Figure S6: Click-CLEM Image of DMSO Control 21](#_Toc195774584)

[Figure S7: Western blot analysis for CuAAC control of CBN-P 22](#_Toc195774585)

[Figure S8: Mass Spectrometric Identification of Target Candidates 23](#_Toc195774586)

[Figure S9: Efficacy of IDH2 Knockdown 23](#_Toc195774587)

[HPLC-Chromatograms for Purity Control of the Target Compound **CBN-P** 24](#_Toc195774588)

[NMR-Spectra of the Target Compounds **CBN-P** 25](#_Toc195774589)

[References 26](#_Toc195774590)

# Unsuccessful CBN-Probe Synthetic Routes

First, synthesis of the CBN-probe **CBN-P-2** was attempted incorporating a published one-pot synthesis of CBN. [1] Unfortunately, this reaction doesn’t tolerate most substrates (Scheme 1-3).

**Scheme S1.** Reactions and conditions: a) 1. citral, BuNH_2_, toluene, reflux, overnight; 2. Dowex50WX8, 30 min, RT; 3. I_2_, toluene, reflux, 5 h; b) 1. LDA, THF, -78°C, 30 min; 2. propargyl bromide, RT, 4 h; c) NH_3_, NH_2_SO_3_H; 2. I_2_ NEt_3_.

**Scheme S2.** Reactions and conditions: a) MOM-Cl, DIPEA, MeCN, RT, overnight; b) NaBH_4_, MeOH, 0°C, 1 h; c) NaH, BnBr, DMF, RT, overnight; d) HCl/MeOH, 50°C; e) 1. citral, BuNH_2_, toluene, reflux, overnight; 2. Dowex50WX8, 30 min, RT; 3. I_2_, toluene, reflux, 5 h; f) Ac_2_O, NaH, DMF; g) H_2_, Pd/C, MeOH; h) CrO_3_, H_2_SO_4_; i) 1. LDA, THF, -78°C, 30 min; 2. propargyl bromide, RT, 4 h; j) 1. NH_3_, NH_2_SO_3_H; 2. I_2_ NEt_3_; k) HCl/MeCN, 60°C.

**Scheme S3.** Reactions and conditions: a) propane-1,3-dithiol, BF_3_ OEt_2_, CH_2_Cl_2_, 0°C 🡪 RT; b) BBr_3_, CH_2_Cl_2_, -70°C 🡪 RT; c) 1. citral, BuNH_2_, toluene, reflux, overnight; 2. Dowex50WX8, 30 min, RT; 3. I_2_, toluene, RT, 5 h; d) MOM-Cl, DIPEA, DMF, RT; e) 1. BuLi, Et_2_O, -78°C; 2. 4-bromo-1-butyne; f) CaCO_3_, MeI, H_2_O, MeCN, 55°C; g) HCl, MeOH, RT; h) 1. NH_3_, HOASA, MeOH, -78°C; 2. NEt_3_, I_2_, CH_2_Cl_2_, RT.

The one-pot synthesis worked out with low yields for the brominated starting material, but the following Grignard reaction (Scheme 5, d) failed.

**Scheme S4.** Reactions and conditions: a) BBr_3_, CH_2_Cl_2_, -78°C 🡪 RT; b) 1. citral, BuNH_2_, toluene, reflux, overnight; 2. Dowex50WX8, 30 min, RT; 3. I_2_, toluene, reflux, 5 h; c) MOM-Cl, DIPEA, DMF, RT; d) 1. Mg, Et_2_O; 2. Pent-1-yn-nitrile, Et_2_O; e) HCl, MeOH, RT; f) 1. NH_3_, HOASA, MeOH, -78°C; 2. NEt_3_, I_2_, CH_2_Cl_2_, RT.

Thioether protection of the ketone enabled the one-pot synthesis, but the low yields in this step and the failed alkylation of the ketone made this synthesis route impossible (Scheme 6).

**Scheme S5.** Reactions and conditions: a) Ethane-1,2-dithiol, BF_3_·Et_2_O, CH_2_Cl_2_, 0 °C → RT; then RT. 16 h; b) 1. citral, BuNH_2_, toluene, reflux, overnight; 2. Dowex50WX8, 30 min, RT; 3. I_2_, toluene, reflux, 5 h; c) AgNO_3_, EtOH/H_2_O, 50 °C, 20 min; d) 1. LDA, THF, -78 °C; 2. propargyl bromide, THF, -79 °C 🡪 RT; e) 1. NH_3_, HOASA, MeOH, -78°C; 2. NEt_3_, I_2_, CH_2_Cl_2_, RT.

Aiming to synthesize probe **CBN-P**, we first tried the one-pot synthesis [1] with phloroglucinol or protected phloroglucinol, but unfortunately that didn’t work out (Scheme 6). Therefore, we used the synthesis route presented in the manuscript to obtain the probe.

**Scheme S6.** Reactions and conditions: a) 1. citral, BuNH_2_, toluene, reflux, overnight; 2. Dowex50WX8, 30 min, RT; 3. I_2_, toluene, reflux, 5 h; b) K_2_CO_3_, Me_2_SO_4_, acetone, 55°C, overnight; c) K_2_CO_3_, benzyl bromide, DMF, RT, 3 h.

# Experimental Procedures

General Information

All reagents were used without further purification and bought from common commercial suppliers in reagent grade. For anhydrous reaction conditions, THF was dried before use by refluxing over sodium slices for at least 2 hours under an argon atmosphere. Thin-layer chromatography was performed on silica gel 60 (alumina foils with fluorescent indicator 254 nm). UV light (254 and 366 nm) was used for detection. For column chromatography, silica gel 60 (particle size 0.040−0.063 mm) was used. Nuclear magnetic resonance (NMR) spectra were recorded with a Bruker AV-400 NMR instrument (Bruker, Karlsruhe, Germany) in a deuterated solvent. Chemical shifts are expressed in parts per million (ppm) relative to the solvent applied (2.50 ppm for ^1^H and 39.5 ppm for ^13^C in DMSO-*d_6_*; 7.26 ppm for ^1^H and 77.2 ppm for ^13^C for CDCl_3_; 2.05 ppm for ^1^H and 206.3 ppm for ^13^C for acetone-*d_6_*; 4.87 ppm for ^1^H and 49.0 ppm for ^13^C for methanol-*d_4_*). The purity of the synthesis products was determined by HPLC (Shimadzu Products), containing a DGU- 20A3R degassing unit, an LC20AB liquid chromatograph, and an SPD-20A UV/vis detector. UV detection was measured at 254 nm. Mass spectra were obtained by an LCMS 2020 (Shimadzu Products). As a stationary phase, a Synergi 4U fusion-RP (150 mm × 4.6 mm) column was used, and as a mobile phase, a gradient of methanol/water with 0.1% formic acid was used. Parameters: A = water, B = methanol, V(B)/(V(A) + V(B)) = from 5 to 95% over 10 min, V(B)/(V(A) + V(B)) = 95% for 5 min, V(B)/(V(A) + V(B)) = from 95 to 5% over 3 min. The method was performed with a flow rate of 1.0 mL/min. Compounds were only used for biological evaluation if the purity was ≥95%. Melting points were determined using an OptiMelt automated melting point system (Scientific Instruments GmbH, Gilching, Germany).

**Scheme S7.** Reactions and conditions: a) 1. LDA, THF, -78°C, 6 h min; 2. Propargyl bromide, rt, overnight; b) Ethylene glycol, triethyl orthoformate, pTsOH, rt, overnight; c) LiAlH_4_, THF, 0°C -> rt, 15 min; d) pTsOH, acetone, rt, 2 h; e) 1. 7 N NH_3_ in MeOH, tBuOCl, tBuOH, rt, 4 h; 2. tBuOCl, tBuOH, rt, 2 h; f) I_2_, PPh_3_, imidazole, CH_2_Cl_2_, rt, 4 h.

Synthesis

*Ethyl 3-oxohept-6-ynoate (****7****).* Ethyl acetoacetate **6** (2.13 mL, 16.9 mmol, 1.0 eq) was dissolved in anhydrous THF (50 mL) and degassed. LDA (2.0M solution, 17.7 mL, 35.4 mmol, 2.1 eq) was added dropwise at -78 °C over 2.5 h, and the resulting mixture was stirred for 2.5 h at -78°C. After dropwise addition of propargyl bromide (80wt% solution in toluene, 2.77 mL, 18.6 mmol, 1.1 eq) over 20 min, the reaction mixture was allowed to reach room temperature and stirred overnight. After addition of sat. NH_4_Cl solution (80 mL), the aqueous phase was extracted with EtOAc (3 x 60 mL). The combined organic phases were washed with 1M HCl sol. (50 mL), H_2_O (50 mL), and brine (50 mL), dried over Na_2_SO_4_, and concentrated under reduced pressure. After column chromatography (SiO_2_, PE/EtOAc 9:1), residual starting material was removed under reduced pressure. The title compound was obtained as yellow oil (2.05 g, 12.2 mmol, 72 %); **^1^H NMR** (400 MHz, CDCl_3_) δ = 4.20 (q, *J* = 7.1 Hz, 2H), 3.46 (s, 2H), 2.81 (t, *J* = 7.0 Hz, 2H), 2.47 (td, *J* = 7.5, 2.6 Hz, 2H), 1.95 (t, *J* = 2.7 Hz, 1H), 1.28 (t, *J* = 7.2 Hz, 4H) ppm; **^13^C NMR** (100 MHz, CDCl_3_) δ = 200.7, 167.0, 82.7, 69.1, 61.6, 49.4, 41.8, 14.2, 13.0 ppm; **MS (ESI, positive)**: *m/z* calc. for [C_9_H_13_O_3_]^+^: 169.09, found: 169.00 [M+H]^+^.

*Ethyl 2-(2-(but-3-yn-1-yl)-1,3-dioxolan-2-yl)acetate (****8****).* Ethyl 3-oxohept-6-ynoate (**7**) (2.05 g, 12.2 mmol, 1.0 eq), ethylene glycol (2.71 mL, 48.6 mmol, 4.0 eq), triethyl orthoformate (4.04 mL, 24.3 mmol, 2.0 eq), and *p*TsOH (209 mg, 1.22 mmol, 0.1 eq) were combined and stirred at room temperature overnight. Sat. NaHCO_3_ (80 mL) was added, and the aqueous phase was extracted with Et_2_O (3 x 50 mL). The organic phase was washed with brine (50 mL), dried over Na_2_SO_4_, and concentrated under reduced pressure. Column chromatography (SiO_2_, PE/EtOAc 6:1) gave the title compound as colourless oil (2.01 g, 9.48 mmol, 78 %); **^1^H NMR** (400 MHz, CDCl_3_) δ = 4.18 – 4.09 (m, 2H), 4.03 – 3.87 (m, 4H), 2.67 – 2.59 (m, 2H), 2.32 – 2.23 (m, 2H), 2.14 – 2.05 (m, 2H), 1.93 – 1.88 (m, 1H), 1.29 – 1.20 (m, 3H) ppm; **^13^C NMR** (100 MHz, CDCl3) δ = 169.2, 108.4, 84.0, 68.2, 65.3, 64.9, 60.7, 42.8, 36.5, 14.3, 12.9 ppm; **MS (ESI, positive)**: *m/z* calc. for [C_11_H_17_O_4_]^+^: 213.11, found: 213.05 [M+H]^+^.

*2-(2-(But-3-yn-1-yl)-1,3-dioxolan-2-yl)ethan-1-ol (****9****)****.*** Ethyl 2-(2-(but-3-yn-1-yl)-1,3-dioxolan-2-yl)acetate (**8**) (2.33 g; 11.0 mmol, 1.0 eq) was dissolved in dry THF (37 mL). LiAlH_4_ (1.25 g, 32.9 mmol, 3.0 eq) was added portionwise at 0 °C over 10 min. The resulting reaction mixture was stirred at room temperature for 15 min and then poured onto ice-water (80 mL) and acidified with 1 M HCl. After filtration, the aqueous phase was extracted with EtOAc (3 x 100 mL), and the organic phases were washed with brine (50 mL) and dried over Na_2_SO_4_. Removing the solvent *in vacuo* gave the title compound as colourless oil (1.82 g, 10.7 mmol, 97 %); **^1^H NMR** (400 MHz, CDCl_3_) δ = 4.05 – 3.94 (m, 4H), 3.79 – 3.72 (m, 2H), 2.32 – 2.23 (m, 2H), 1.97 – 1.90 (m, 5H) ppm; **^13^C NMR** (100 MHz, CDCl_3_) δ = 111.2, 84.1, 68.4, 65.1 (2C), 58.9, 38.4, 36.1, 13.3 ppm; **MS (ESI, positive)**: *m/z* calc. for [C_9_H_15_O_5_]^+^: 171.10, found: 171.05 [M+H]^+^.

*1-Hydroxyhept-6-yn-3-one (****10****).* To a stirred solution of 2-(2-(but-3-yn-1-yl)-1,3-dioxolan-2-yl)ethan-1-ol (**9**) (932 mg, 5.48 mmol, 1.0 eq) in acetone was added *p*-toluenesulfonic acid (236 mg, 1.37 mmol, 0.25 eq). The reaction mixture was stirred at room temperature for 4 h, then quenched upon addition of brine (80 mL). The mixture was extracted with EtOAc (4 x 70 mL). The combined organic phases were dried over Na_2_SO_4_, and the solvents were removed under reduced pressure to give the title compound as colourless oil (653 mg, 5.18 mmol, 95 %); **^1^H NMR** (400 MHz, CDCl_3_) δ = 3.87 (t, *J* = 5.4 Hz, 2H), 2.74 – 2.66 (m, 4H), 2.47 (td, *J* = 7.1, 2.6 Hz, 2H), 1.96 (t, *J* = 2.6 Hz, 1H) ppm; **^13^C NMR** (100 MHz, CDCl_3_) δ = 209.2, 82.9, 69.1, 57.9, 44.7, 42.0, 13.0 ppm; **MS (ESI, positive)**: *m/z* calc. for [C_9_H_15_O_5_]^+^: 127.08, found: 127.10 [M+H]^+^.

t-*Butyl hypochlorite.* Sodium hypochlorite solution (10wt%, 120 mL), was cooled below 2 °C, then acetic acid (8.4 mL) and *t*-butanol (15.6 mL, 163 mmol) were added. The resulting mixture was stirred for 25 min in an ice bath, then put into a separation funnel. Phases were separated and the organic phase was washed with H_2_O (2 x 10 mL) and dried over Na_2_SO_4_ to give the title compound as yellow liquid (3.27 g, 30.1 mmol, 18 %). The compound was used without further purification or characterization.

*2-(3-(But-3-yn-1-yl)-3H-diazirin-3-yl)ethan-1-ol (****11****).* To a stirred solution of 1-hydroxyhept-6-yn-3-one (**10**) (49 mg, 388 µmol, 1.0 eq) in 7N NH_3_ in methanol (577 µL, 10.4 eq) was added *t*-BuOCl (132 µL, 1.17 mmol, 3.0 eq) in *t*-BuOH (500 µL) dropwise. The resulting reaction mixture was stirred for 4 h at room temperature. Then, excess NH_3_ was removed by bubbling N_2_ through the mixture. After addition of added *t*-BuOCl (66 µL, 583 µmol, 1.5 eq) in *t*-BuOH (250 µL), the reaction was stirred for an additional hour at room temperature. The reaction was quenched upon addition of sat. Na_2_S_2_O_3_ (30 mL) and extracted with Et_2_O (3 x 25 mL). The combined organic phases were washed with brine (30 mL), dried over Na_2_SO_4_, and the solvents were removed under reduced pressure. Subsequent column chromatography (SiO_2_, CH_2_Cl_2_/Et_2_O 95:5) gave the title compound as colourless oil (27 mg, 195 µmol, 50 %); **^1^H NMR** (400 MHz, CDCl_3_) δ = 3.49 (t, *J* = 6.2 Hz, 2H), 2.09 – 1.97 (m, 3H), 1.76 – 1.63 (m, 4H), 1.54 (s, 1H) ppm; ^13^C NMR (101 MHz, CDCl_3_) δ 83.0, 69.4, 57.5, 35.7, 32.8, 26.7, 13.4 ppm; **MS (ESI, positive)**: *m/z* calc. for [C_7_H_11_N_2_O]^+^: 139.09, found: 139.10 [M+H]^+^.

*3-(But-3-yn-1-yl)-3-(2-iodoethyl)-3*H*-diazirine (****5****).* I_2_ (87 mg, 630 µmol, 1.2 eq) was added to a stirred solution of imidazole (129 mg, 1.89 mmol, 3.0 eq) and PPh_3_ (182 mg, 693 µmol, 1.1 eq) in CH_2_Cl_2_ (3.8 mL) at 0 °C. The mixture was stirred for 10 min at 0 °C, then a solution of 2-(3-(but-3-yn-1-yl)-3*H*-diazirin-3-yl)ethan-1-ol (**11**) (87 mg, 630 µmol, 1.0 eq) in CH_2_Cl_2_ was added dropwise at 0 °C. The reaction was stirred for 4.5 h at room temperature in the dark, then quenched upon addition of sat. Na_2_S_2_O_3_. The aqueous phase was extracted with CH_2_Cl_2_. The combined organic phases were washed with brine dried over Na_2_SO_4_ and concentrated *in vacuo*. A short filter column (SiO_2_, PE 🡪 PE/Et_2_O 10:1) gave the title compound as colourless liquid (122 mg, 492 µmol, 78 %); **^1^H NMR** (400 MHz, CDCl_3_) δ = 2.89 (t, *J* = 7.6 Hz, 2H), 2.12 (t, *J* = 7.6 Hz, 2H), 2.06 – 1.99 (m, 3H), 1.68 (t, *J* = 7.1 Hz, 2H) ppm; **^13^C NMR** (100 MHz, CDCl_3_) δ = 82.6, 69.6, 37.7, 32.0, 28.8, 13.4, -3.9 ppm.

*(1'R,2'R)-5'-methyl-2'-(prop-1-en-2-yl)-1',2',3',4'-tetrahydro-[1,1'-biphenyl]-2,4,6-triol (****3****).* Phloroglucinol **2** (4.14 g, 32.8 mmol, 10.0 eq) was dissolved in dry THF (20 mL) and cooled to -10°C. Then, BF_3_·OEt_2_ (40.5 µL, 328 µmol, 0.1 eq) was added, followed by a solution of (1s,4s)-1-methyl-4-(prop-1-en-2-yl)cyclohexan-1-ol **1** (500 mg, 3.28 mmol, 1.0 eq) in dry THF (5 mL). The resulting reaction mixture was stirred at -10°C 🡪 0°C for 3 h and then quenched upon addition of sat. NaHCO_3_ (50 mL). The aqueous phase was extracted with EtOAc (3 x 40 mL). The combined organic phases were washed with brine (50 mL), dried over Na_2_SO_4_, and concentrated *in vacuo*. Subsequent column chromatography (SiO_2_, PE/EtOAc 2:1) gave the title compound in quantitative yield as slightly orange oil (853 mg, 3.28 mmol, quant.); **^1^H NMR** (400 MHz, CDCl_3_) δ = 6.07 (s, 1H), 6.00 – 5.87 (m, 2H), 5.54 (s, 1H), 5.09 (s, 2H), 4.66 (s, 1H), 4.56 (s, 1H), 3.83 – 3.74 (m, 1H), 2.35 (td, *J* = 10.8, 3.7 Hz, 1H), 2.26 – 2.16 (m, 1H), 1.82 – 1.73 (m, 5H), 1.65 (s, 3H) ppm; **^13^C NMR** (100 MHz, CDCl_3_) δ = 155.4 (2C), 149.4, 149.3, 140.2, 124.3, 114.7, 110.9 (2C), 109.2, 46.4, 37.0, 30.4, 28.4, 23.7, 20.5 ppm; **MS (ESI, positive)**: *m/z* calc. for [C_16_H_21_O_3_]^+^: 261.15, found: 261.05 [M+H]^+^.

*6,6,9-Trimethyl-6*H*-benzo[*c*]chromene-1,3-diol (****4****).* (1'*R*,2'*R*)-5'-methyl-2'-(prop-1-en-2-yl)-1',2',3',4'-tetrahydro-[1,1'-biphenyl]-2,4,6-triol (**3**) (960 mg, 3.69 mmol, 1.0 eq) and I_2_ (2.81 g, 11.1 mmol, 3.0 eq) were combined in dry toluene (35 mL) and stirred at reflux overnight. The reaction was quenched upon addition of sat. Na_2_S_2_O_3_ solution (300 mL) and extracted with EtOAc (3 x 100 mL). The organic phases were washed with brine (100 mL), dried over Na_2_SO_4_ and the solvents were removed *in vacuo*. Column chromatography (SiO_2_, PE/EtOAc 4:1) gave the title compound as brown oil (154 mg 604 µmol, 16 %); **^1^H NMR** (400 MHz, MeOD) δ = 8.26 (d, *J* = 1.8 Hz, 1H), 7.09 (d, *J* = 7.9 Hz, 1H), 6.98 – 6.93 (m, 1H), 6.04 (d, *J* = 2.4 Hz, 1H), 5.93 (d, *J* = 2.4 Hz, 1H), 2.32 (s, 3H), 1.53 (s, 6H) ppm; **^13^C NMR** (100 MHz, MeOD) δ = 159.2, 157.7, 156.8, 137.3, 136.8, 129.7, 127.5, 127.2, 123.2, 104.8, 98.1, 97.7, 78.2, 27.4, 21.6 ppm; **MS (ESI, positive)**: *m/z* calc. for [C_16_H_17_O_3_]^+^: 257.12, found: 257.00 [M+H]^+^.

*3-(2-(3-(But-3-yn-1-yl)-3*H*-diazirin-3-yl)ethoxy)-6,6,9-trimethyl-6*H*-benzo[*c*]chromen-1-ol (****CBN-P****).* 6,6,9-Trimethyl-6*H*-benzo[*c*]chromene-1,3-diol (**4**) (24 mg, 93.6 µmol, 2.0 eq) was dissolved in dry acetone (300 µL) and K_2_CO_3_ (13 mg, 93.6 µmol, 2.0 eq) was added. The reaction mixture was stirred at room temperature for 30 min. Then, 3-(but-3-yn-1-yl)-3-(2-iodoethyl)-3*H*-diazirine (**5**) (6.87 µL, 46.8 µmol, 1.0 mmol) was added and the mixture was stirred at 50 °C overnight. After addition of 1M HCl (15 mL), the mixture was extracted with EtOAc (3 x 10 mL). The combined organic phases were washed with brine (10 mL), dried over Na_2_SO_4_, and the solvents removed *in vacuo*. Preparative TLC (SiO_2_, PE/EtOAc 9:1) gave the title compound as light brown oil (1 mg, 2.66 µmol, 6 %); **^1^H NMR** (600 MHz, CDCl_3_) δ = 8.09 – 8.07 (m, 1H), 7.14 (d, *J* = 7.8 Hz, 1H), 7.06 – 7.04 (m, 1H), 6.15 (d, *J* = 2.5 Hz, 1H), 6.07 (d, *J* = 2.5 Hz, 1H), 3.80 (t, *J* = 6.2 Hz, 2H), 2.38 (s, 3H), 2.07 (td, *J* = 7.5, 2.6 Hz, 2H), 2.00 (t, *J* = 2.7 Hz, 1H), 1.87 (t, *J* = 6.2 Hz, 2H), 1.74 (t, *J* = 7.6 Hz, 2H), 1.59 (s, 6H) ppm; **^13^C NMR** (150 MHz, CDCl_3_) δ = 159.2, 156.0, 154.3, 137.2, 136.3, 127.5, 127.4, 125.8, 122.8, 105.1, 97.5, 96.8, 82.9, 77.9, 69.4, 62.7, 33.0, 32.8, 29.9, 27.2, 21.7, 13.5 ppm; **MS (ESI, positive)**: *m/z* calc. for [C_23_H_25_N_2_O_3_]^+^: 377.19, found: 377.10 [M+H]^+^.

Cell culture general procedures

HT22 cells were grown in Dulbecco’s Modified Eagle Medium (DMEM, Sigma Aldrich, Munich, Germany) supplemented with 10% (v/v) fetal calf serum (FCS) and 1% (v/v) penicillin-streptomycin. Cells were subcultured every two days and incubated at 37°C with 5% CO_2_ in a humidified incubator.

Compounds were dissolved in dimethyl sulfoxide (DMSO, Sigma Aldrich, Munich, Germany) as stock solutions and diluted further into culture medium.

For determination of cell viability, a colorimetric 3-(4,5-dimethylthiazol-2-yl)-2,5- diphenyl tetrazolium bromide (MTT, Sigma Aldrich, Munich, Germany) assay was used. MTT solution (5 mg/mL in PBS) was diluted 1:10 with medium and added to the wells after removal of the old medium. Cells were incubated for 3 h and then lysis buffer (10% SDS) was applied. The next day, absorbance at 560 nm was determined with a multiwell plate photometer (Tecan, SpectraMax 250).

*Neurotoxicity and oxytosis in HT22 cells.* 5x10^3^ cells per well were seeded into sterile 96-well plates and incubated overnight. For the neurotoxicity assay, medium was removed and 1.25, 2.5, 5 or 10 µM of the compound diluted with medium from a 10 mM stock solution was added to the wells. 0.05% DMSO in DMEM served as a control. Cells were incubated for 24 h when neurotoxicity was determined using a colorimetric MTT assay.

For the oxytosis assay, 5 mM glutamate (monosodium-*L*-glutamate, Sigma Aldrich, Munich, Germany) solely or together with 1.25, 2.5, 5 or 10 µM of the respective compounds were added to the cells and incubated for 24 h. After 24 h incubation, cell viability was determined using a colorimetric MTT assay as described above. Results are presented as percentage of untreated control cells. Data is expressed as means ± SEM of three independent experiments. Analysis was accomplished using GraphPad Prism 10 Software applying Oneway ANOVA followed by Dunnett’s multiple comparison posttest. Levels of significance: * p < 0.05; ** p < 0.01; *** p < 0.001; **** p < 0.0001.

*Ferroptosis in HT22 cells.* 3x10^3^ cells per well were seeded into sterile 96-well plates and incubated overnight. The next day medium was exchanged with fresh medium and 100 nM RSL3 was added with vehicle (DMSO) to induce oxidative stress, or together with 0.15, 0.32, 0.625, 1.25, 2.5, 5, 10 or 20 µM of the respective compound for protection. After 24 h cell viability was determined using a colorimetric MTT assay. Results are presented as percentage of untreated control cells. Data are expressed as means ± SEM of three independent experiments. Analysis was accomplished using GraphPad Prism 10 Software applying Oneway ANOVA followed by Dunnett’s multiple comparison posttest. Levels of significance: * p < 0.05; ** p < 0.01; *** p < 0.001; **** p < 0.0001.

*ATP Depletion in HT22 Cells.* 3x10^3^ cells per well were seeded into sterile 96-well plates and incubated overnight. The next day, medium was exchanged with fresh medium. Iodoacetic acid (IAA, 17.5 μM) was added with vehicle (DMSO) as a negative control, or together with 1.25, 2.5, 5, or 10 μM concentrations of the respective compound for protection. After 2 h of incubation at 37 °C, the medium was aspirated, and fresh medium was applied; only the compounds at the same respective concentrations were added without IAA. After 24 h, cell viability was determined using the MTT assay. Results are presented as percentages of untreated control cells. Data are expressed as means ± SEM of three independent experiments. The analysis was accomplished using GraphPad Prism 10 Software applying One-way ANOVA followed by Dunnett’s multiple comparison posttest. Levels of significance: * p < 0.05; ** p < 0.01; *** p < 0.001; **** p < 0.0001.

Western blots

*Sample preparation.* 2x10^5^ HT22 cells per 60 mm dish were grown overnight before treatment with the respective compounds at the indicated concentrations and incubation times. For whole lysate analysis cells were washed twice with PBS, scraped into 50 µL lysis buffer (10 mM Tris, pH 7.4, 100 mM NaCl, 1 mM EDTA, 1 mM EGTA, 1 mM NaF, 20 mM Na4P2O7, 2 mM Na3VO4, 1% Triton X-100, 10% glycerol, 0.1% SDS, 0.5% deoxycholate, 1x protease inhibitor cocktail and 1x phosphatase inhibitor cocktail) and incubated on ice for 30 min. After centrifugation (14000 rpm, 4 min, 4 °C), the supernatant was transferred and protein concentrations were quantified by the bicinchoninic acid assay (Pierce, Rockford, IL, USA). Samples were adjusted to equal protein concentrations, 5x Western blot sample buffer (74 mM Tris-HCl pH 8.0, 6.25% SDS, 10% β-mercaptoethanol, 20% glycerol) was added to a final concentration of 2.5x and samples were sonicated briefly and boiled for 5 min.

*Western blotting.* Equal amounts of protein (10-20 µg) per lane were used for SDS-PAGE. Proteins were separated using 4-20% Criterion™ TGX Stain-Free™ Protein Gels (Bio-Rad, Hercules, CA, USA) and subsequently were transferred to PVDF membranes. Membranes were blocked with 5% skim milk in TBS-T (20 mM Tris buffer pH 7.5, 0.5 M NaCl, 0.05% Tween 20) for 1 h at room temperature and incubated overnight at 4 °C with the primary antibody diluted in 5% BSA in TBS/0.05% Tween 20. HRP-conjugated rabbit anti-actin (#5125, 1:10 000), anti-OPA1 (#80471, 1:3000), anti-MFN2 (#9482, 1:1000), anti-DRP1 (#8570, 1:3000), anti-MFF (#84580, 1:3000), anti-TOM20 (#42406, 1:3000), anti-SIRT1 (#9475, 1:3000), anti-MCU (#14997, 1:3000), anti-IDH2 (#56439, 1:1000) from Cell Signaling Technology (Danvers, MA, USA); anti-GPX4 (#sc-166570, 1:1000) from Santa Cruz Biotechnology Inc. (Dallas, TX, USA) were used as primary antibodies. Subsequently, blots were washed in TBS/0.05% Tween 20 and incubated for 1 h at room temperature in horseradish peroxidase goat anti-rabbit or goat anti-mouse (#1721019 and # 1721011 Bio-Rad, Hercules, CA, USA) diluted 1/5000 in 5% skim milk in TBST. After additional washing, protein bands were detected by chemiluminescence using the SuperSignal West Pico PLUS Chemiluminescent Substrate (Pierce). Autoradiographs were scanned using a ChemiDoc MP Imaging System (Bio-Rad, Hercules, CA, USA). Band density was measured using Image Lab software (Bio-Rad, Hercules, CA, USA). Each Western blot was repeated at least three times with independent protein samples. Band density was normalized to actin or total protein in each lane.

Reactive Oxygen Species Measurements

5x10^3^ HT22 cells per well were seeded onto 96-well black walled plates and incubated overnight. After the desired treatment, mitochondrial superoxide ROS were detected with MitoSOX Red reagent (Ex/Em = 510/580 nm). The experiment was performed according to the manufacturer’s instructions. Fluorescence was measured on a SpectraMax M5 microplate reader. Data were normalized for total protein/well. Each condition was analyzed in sixteen wells per independent experiment (n = 16). Results were verified by live-cell imaging under a fluorescence microscope.

Lipid Peroxidation Measurements

5x10^3^ HT22 cells per well were seeded onto 96-well black walled plates and incubated overnight. After the desired treatments, cells were labeled with 2.5 µM C11-BODIPY 581/591 (oxidized form Ex/Em = 488/520 nm) at 37 °C for 2 h. Experiments were performed according to the manufacturer’s instructions. Fluorescence was measured on a SpectraMax M5 microplate reader. Data were normalized for total protein/well. Each condition was analyzed in sixteen wells per independent experiment (n = 16). Results were verified by live-cell imaging under a fluorescence microscope.

Calcium Measurements

5x10^3^ HT22 cells per well were seeded onto 96-well black walled plates and incubated overnight. After the desired treatments, Ca2+ levels were detected with Fluo-4 AM (Ex/Em = 494/516 nm) and Rhod-2 AM (Ex/Em = 552/581 nm) calcium indicator dyes specific to cytosol and mitochondria, respectively. Fluorescence was measured on a SpectraMax M5 microplate reader. Experiments were performed according to the manufacturer’s instructions. Data were normalized for total protein/well. Each condition was analyzed in sixteen wells per independent experiment (n = 16). Results were verified by live-cell imaging under a fluorescence microscope.

Seahorse XF Analysis

The assay procedure was previously described.[2] Cellular oxygen consumption rates (OCR) were assayed with a XF Cell Mito Stress Test Kit using a Seahorse XFe96 Extracellular Flux Analyzer (Seahorse Bioscience, North Billerica, MA). Complete Seahorse XF DMEM assay medium was supplemented with 10 mM glucose, 1 mM pyruvate and 2 mM L-glutamine, at pH 7.4. Mitochondrial ETC inhibitors were used at the following concentrations: 1.5 μM oligomycin, 2 μM FCCP, and 0.5 μM of a 1:1 mixture of rotenone and antimycin A. Analyses were conducted using Wave software and XF Report Generators (Agilent Technologies). The sensor cartridge for the XFe analyzer was hydrated overnight at 37 °C before the experiment. OCR data were normalized for total protein/well. Each condition was analyzed in 16 wells per independent experiment (n = 16).

For HT22 cells, 2x10^3^ cells/well were seeded onto the Seahorse XFe96 plates under normal culture condition as described above. The next day, cells were pretreated with the compounds at the desired concentrations and RSL3 at 150 nM and incubated for 16 h. Immediately before the assay, the culture medium in the plates was replaced with complete Seahorse XF DMEM assay medium, supplemented with compounds and RSL3 at the same concentrations. The plates were incubated for 1 h at 37 °C prior to the XF Cell Mito Stress tests according to the manufacturer’s instructions.

Fluorescence microscopy

*CuAAC in fixed cells for fluorescence microscopy.* 4x10^4^ HT22 cells stably expressing a mitochondria-targeted GFP (mito-GFP) were grown on 24 well plates with coverslips. The next day, compounds were added in indicated concentrations and incubated for 30 min or overnight at 37 °C. After washing 2 times for 10 min with warm PBS, cells were fixed with freshly prepared 4% paraformaldehyde (PFA) in PBS at 37 °C for 20 min and permeabilized with 0.1% Triton X-100 in PBS at room temperature for 15 min. The CuAAC reaction mixture was prepared in an Eppendorf tube by mixing Cy3-azide (20 µM final concentration), freshly prepared tris(2-carboxyethyl)phosphine hydrochloride (TCEP) (1 mM final concentration), tris[(1-benzyl-1H-1,2,3-triazol-4-yl)methyl amine (TBTA) (0.1 mM final concentration) and copper sulfate (1 mM final concentration) in PBS. The CuAAC reaction mixture was added to the permeabilized cells and incubated for 1 h at room temperature in the dark. After washing with PBS, nuclei were co-stained with DAPI using NucBlue™ Fixed Cell ReadyProbes™ Reagent (Invitrogen, Carlsbad, CA, USA) Three additional PBS washing steps were rendered prior to placing the coverslips on slides with mounting media. Images were acquired using a Zeiss LSM 880 Rear Port Laser Scanning Confocal and Airyscan FAST Microscope. DAPI, GFP and Cy3 were excited sequentially using 405, 488 and 561 laser lines, respectively and collected using the corresponding filters and the Airyscan detector. Final analysis of the images was performed using ImageJ software.

*Click-CLEM Experiment.* For the click-CLEM experiment, HT22 cells were grown in 100 mm dishes to confluency. Cells were treated with 5 µM CBN-Por 1 µM DMSO for 30 min at 37 °C. Afterwards, cells were rinsed twice with PBS, then scraped into 500 µL PBS. After centrifugation (1100 rpm, 4 min), the supernatant was removed, and the cell pellet was resuspended in 4 % PFA + 0.05 % glutaraldehyde in PBS and fixed for 15 min at 37 °C with shaking. Cells were then permeabilized with 0.5% Triton X-100 in PBS at room temperature for 15 min with shaking. CuAAC was performed on a cell suspension with the Click-iT™ Plus Alexa Fluor™ 488 Picolyl Azide Toolkit (Invitrogen, Carlsbad, CA, USA) according to the manufacturer’s procedure with 5 µM AF488. Afterwards, the cellular pellet was washed 4 x with PBS, then 4 % PFA + 0.05 % glutaraldehyde in PBS were added. After centrifugation (13500 rpm, 5 min), the cellular pellet was fixed overnight at 4 °C in the dark. The next day, the pellet was washed 3 x with cold PBS and incubated in ammonium chloride (50 mM ammonium chloride in PBS) for 15 min. Following several washes in water, the samples were embedded in LR White resin according to the following Progressive Lowering of Temperature (PLT) protocol. The initial dehydration step of 30% ethanol in water happens on ice (2 × 15 min), then the following steps (50%, 70% (with 0.2% uranyl acetate), 90% (with 0.2% uranyl acetate) and 100% ethanol; 2 times 30 min each) were performed at -20 °C. A mixture of 100% ethanol and LR White (1:1) was incubated overnight and resin infiltration was performed within 24 h, with washes after 1 h, 4 h, 20 h and 24 h at 4 °C. The resin was polymerized at 42 °C for 3 days. 100 nm sections were produced using a special diamond knife with a boat large enough to accommodate glass slides (histo Jumbo diamond knife, DiATOME). Slides were submerged in the boat before sectioning.[3] Then the desired number of sections was cut without interruption. Ultra-thin sections were stained with Methyl Green (1:10000) for 30 min[4] rinsed off and mounted in Mowiol and high precision coverslips. Fluorescence images were acquired using an ELYRA S.1 super-resolution structured illumination microscope (Zeiss) and channel alignment was performed using TetraSpeck^TM^ fluorescent beads. The samples were then processed for imaging in the SEM by removing the coverslip, washing off the mounting medium and contrasting with heavy metals as follows: the sections are first incubated in 2.5% uranyl acetate in ethanol for 15 min and in 50% Reynolds’ lead citrate in H_2_O for 10 min.[5] Carbon coating was performed with the Compact Coating Unit CCU-010 (Safematic, Bad Ragaz, Switzerland) resulting in a ca. 6 nm coating. Images were taken with the JSM-7500F scanning electron microscope (JEOL Germany, GmbH, Freising, Germany). Registration of the fluorescence and electron microscopy images was accomplished with Inkscape 0.91 (Inkscape Community) using the heterochromatin pattern as an intrinsic landmark in both channels. For an unbiased correlation the channel of interest (AF488 signal) was hidden and only the DNA signal (Methyl Green) was used. After the correlation the channel of interest was revealed, and all channels could be merged. More details on the procedure can be found in Markert *et al.*.[3]

CuAAC with Cy3-Azide

2×10^5^ Cells were grown in 60 mm dishes overnight. Cells were pre-treated with CBN at the indicated concentration for 1 h. CBN-P or DMSO were added at the indicated concentration and incubated for 1 h, 2 h, or 4 h. The cells were washed with ice-cold PBS and irradiated with 365 nm for 30 min on ice. Cells were then scraped into 60 μL of lysis buffer, sonicated for 10s, centrifuged at 14 000 rpm for 40 min at 4 °C to pellet cellular debris. The supernatant was transferred to fresh Eppendorf tubes, and the protein concentration was determined by the bicinchoninic acid method (Pierce). The CuAAC reaction was performed in a final volume of 120 μL. Therefore, 100 μg of protein were adjusted to the appropriate volume with PBS, and 20 μM Cy3-azide was added followed by freshly prepared tris(2-carboxyethyl)phosphine hydrochloride (TCEP) (1 mM final concentration), tris[(1-benzyl-1H-1,2,3-triazol-4-yl)methylamine (TBTA) (0.1 mM final concentration), and copper sulfate (1 mM final concentration). The mixture was vortexed and incubated rotating at room temperature for 1 h in the dark after which the reaction was quenched by the addition of 120 μL of 5X sample buffer. Samples were sonicated for 5 s and boiled for 5 min. Then, the samples were submitted to SDS−PAGE, transferred to a nitrocellulose membrane, and examined according to the Western blot protocol described above using fluorescence analysis for Cy3-signal detection (555 nm/566 nm).

CuAAC with HT22 Cells and Affinity Purification of Target Proteins

HT22 cells were grown in 10 cm dishes to 90% confluency. Cells were pre-treated with CBN (120 µM) for 30 min. CBN-P (60 μM) or DMSO were added and incubated for 1 h. The cells were washed with ice-cold PBS and irradiated with 365 nm for 30 min on ice. Cells were then scraped into 350 μL of lysis buffer (TBS with 1% NP-40, pH 7.4) and left shaking for 20 min at 4 °C. Lysates were centrifuged at 14 000 rpm for 20 min at 4 °C to pellet cellular debris. The supernatant was transferred to fresh Eppendorf tubes, and the protein concentration was determined by the bicinchoninic acid method (Pierce).

The CuAAC reaction was conducted in an Eppendorf tube in a final volume of 500 μL. Protein (2000 μg) was adjusted to the needed volume with TBS and 150 μM Azide-SS-Biotin (BroadPharm, San Diego, CA, USA); 1 mM freshly prepared tris(2-carboxyethyl)-phosphine hydrochloride, 0.1 mM tris[(1-benzyl-1H-1,2,3-triazol-4-yl)methylamine, and 1 mM copper sulfate were added. The reaction mixture was vortexed, and then the reaction proceeded for 3 h at room temperature with gentle shaking at which time a precipitate formed. After centrifugation at 6500 g for 4 min at 4 °C, the supernatant was removed. The pellet was washed with 500 μL of ice-cold methanol and sonicated for 4 s, and after 10 min incubation rotating at 4 °C, the suspension was centrifuged at 6500 g for 4 min at 4 °C. The supernatant was removed, and the washing step was repeated once. For solubilization, 200 µL of 1.2% SDS in PBS was added to the pellet after the second washing step, and it was sonicated for 5 s and boiled for 5 min when the sample was diluted with 1 mL PBS to a final SDS concentration of 0.2%. For affinity purification, streptavidin magnetic beads (#S1420S, New England Biolabs) were used according to the manufacturer’s protocol with an extended incubation time of 2 h for proteome incubation. After elution with 50 µL TCEP (20 mM) for 1 h at 37 °C, the proteins were precipitated with 200 µL pre-chilled acetone overnight at -20 °C. After centrifugation (12,000 g, 20 min, 4 °C), the supernatant was removed, and the pellet was washed once with 500 µL ice-cold acetone. Then, the pellet was left to dry.

Single-pot, solid-phase-enhanced sample preparation (SP3)

Samples were processed using an adapted SP3 protocol.[6] Briefly, 250µl reconstitution solution was added to the aceton precipitated samples. DTT was added to a concentration of 5mM. Alkylation was performed with 20mM iodoacetamide. 10mM additional DTT was used for quenching. Equal volumes of two types of Sera-Mag Speed Beads (Cytiva, #45152101010250 and #65152105050250) were combined, washed with water and 10 µL of the bead mix were added to each sample. 260µl 100% ethanol was added and samples were incubated for 5min at 24°C, 1000rpm. Beads were captured on a magnetic rack for 2 min, and the supernatant was removed. Beads were washed twice with 200µl 80% ethanol (Chromasolv, Sigma) and then once with 1000µl 80% ethanol. Digest was performed on beads with 0.25µg Trypsin (Gold, Mass Spectrometry Grade, Promega) and 0.25 µg Lys-C (Wako) in 100µl 100mM ammonium bicarbonate at 37°C overnight. Peptides were desalted using C-18 Stage Tips.[7] Each Stage Tip was prepared with three discs of C-18 Empore SPE Discs (3M) in a 200 µl pipette tip. Peptides were eluted with 60 % acetonitrile in 0.1 % formic acid, dried in a vacuum concentrator (Eppendorf), and stored at -20 °C. Peptides were dissolved in 2 % acetonitrile / 0.1 % formic acid prior to nanoLC-MS/MS analysis.

NanoLC-MS/MS analysis

NanoLC-MS/MS analyses were performed on an Orbitrap Fusion (Thermo Scientific) equipped with a PicoView Ion Source (New Objective) and coupled to an EASY-nLC 1000 (Thermo Scientific). Peptides were loaded on a trapping column (2 cm x 150 µm ID, PepSep) and separated on a capillary column (30 cm x 150 µm ID, PepSep) both packed with 1.9 µmC18 ReproSil and separated with a 120-minute linear gradient from 3% to 30% acetonitrile and 0.1% formic acid and a flow rate of 500 nl/min. Both MS and MS/MS scans were acquired in the Orbitrap analyzer with a resolution of 60,000 for MS scans and 30,000 for MS/MS scans. HCD fragmentation with 35 % normalized collision energy was applied. A Top Speed data-dependent MS/MS method with a fixed cycle time of 3 s was used. Dynamic exclusion was applied with a repeat count of 1 and an exclusion duration of 90 s; singly charged precursors were excluded from selection. Minimum signal threshold for precursor selection was set to 50,000. Predictive AGC was used with AGC a target value of 4x10^5^ for MS scans and 5x10^4^ for MS/MS scans. EASY-IC was used for internal calibration.

MS data analysis

Raw MS data files were analyzed with MaxQuant version 1.6.2.2.[8] Database search was performed with Andromeda, which is integrated in the utilized version of MaxQuant. The search was performed against the UniProt Mouse Reference Proteome database (Release2024_01, UP000000589, 63191 entries). Additionally, a database containing common contaminants was used. The search was performed with tryptic cleavage specificity with 3 allowed miscleavages. Protein identification was under control of the false-discovery rate (FDR; <1% FDR on protein and peptide spectrum match (PSM) level). In addition to MaxQuant default settings, the search was performed against following variable modifications: Protein N-terminal acetylation, Gln to pyro-Glu formation (N-term. Gln) and oxidation (Met). Carbamidomethyl (Cys) was set as fixed modification. Further data analysis was performed using R scripts developed in-house. LFQ intensities were used for protein quantitation.[9] Proteins with less than two razor/unique peptides were removed. Missing LFQ intensities in the control samples were imputed with values close to the baseline. Data imputation was performed with values from a standard normal distribution with a mean of the 5% quantile of the combined log10-transformed LFQ intensities and a standard deviation of 0.1.

For the identification of significantly enriched proteins, median log2 transformed protein ratios were calculated from the two replicate experiments and boxplot outliers were identified in intensity bins of at least 300 proteins. Log2 transformed protein ratios of sample versus control with values outside a 1.5x (significance 1) or 3x (significance 2) interquartile range (IQR), respectively, were considered as significantly enriched in the individual replicates.

Transfection

HT22 cells were plated at 5×10^5^ cells per dish in 60 mm tissue culture dishes and grown overnight. The cells were then transfected with 166 pmol siRNA (IDH2 #sc-62488 from Santa Cruz Biotechnology) or control siRNA (no. 1027280; Qiagen) using RNAi max (Invitrogen) according to the manufacturer’s instructions. After growth overnight, the cells were subcultured for analysis, as described above.

# Figure S1: NOESY of CBN-P


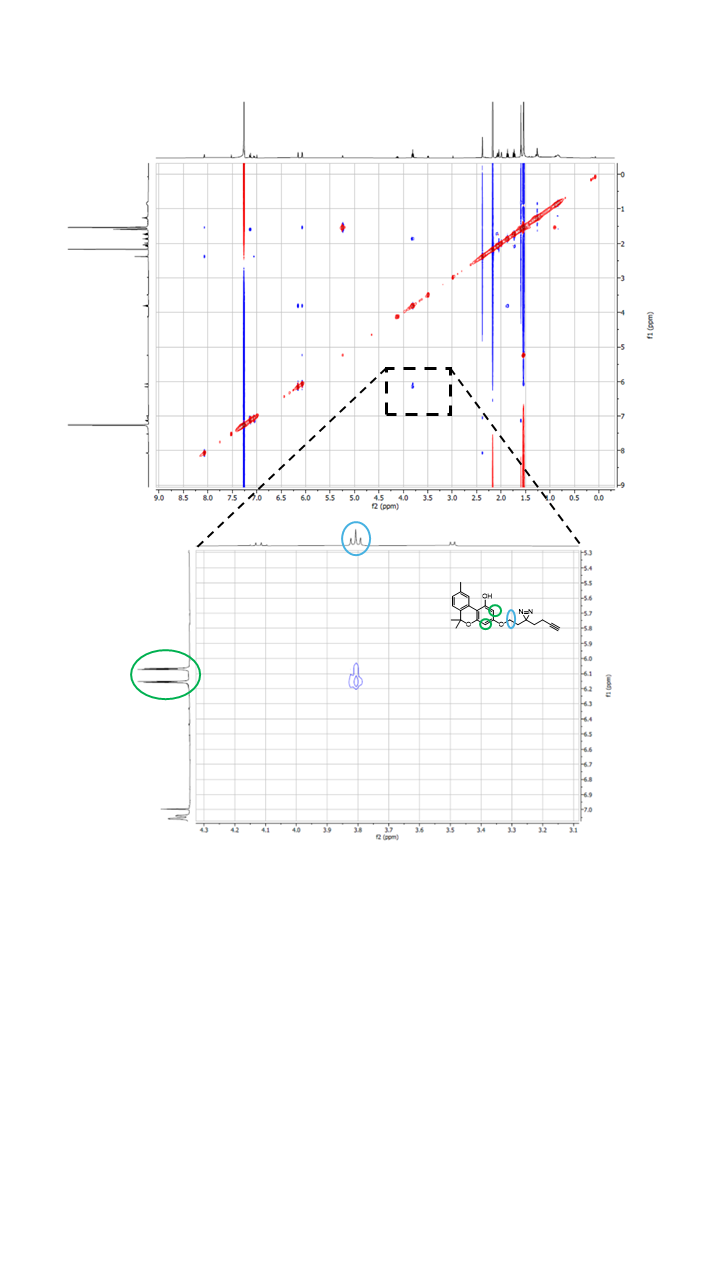


**Figure S1.** HH-NOESY spectrum of CBN-P with enlarged area of interest. The enlarged area shows spatial proximity of the marked protons (CH_2_-group of the linker and CH of the phenol derivative).

# Figure S2: Toxicity of CBN-P on HT22 cells


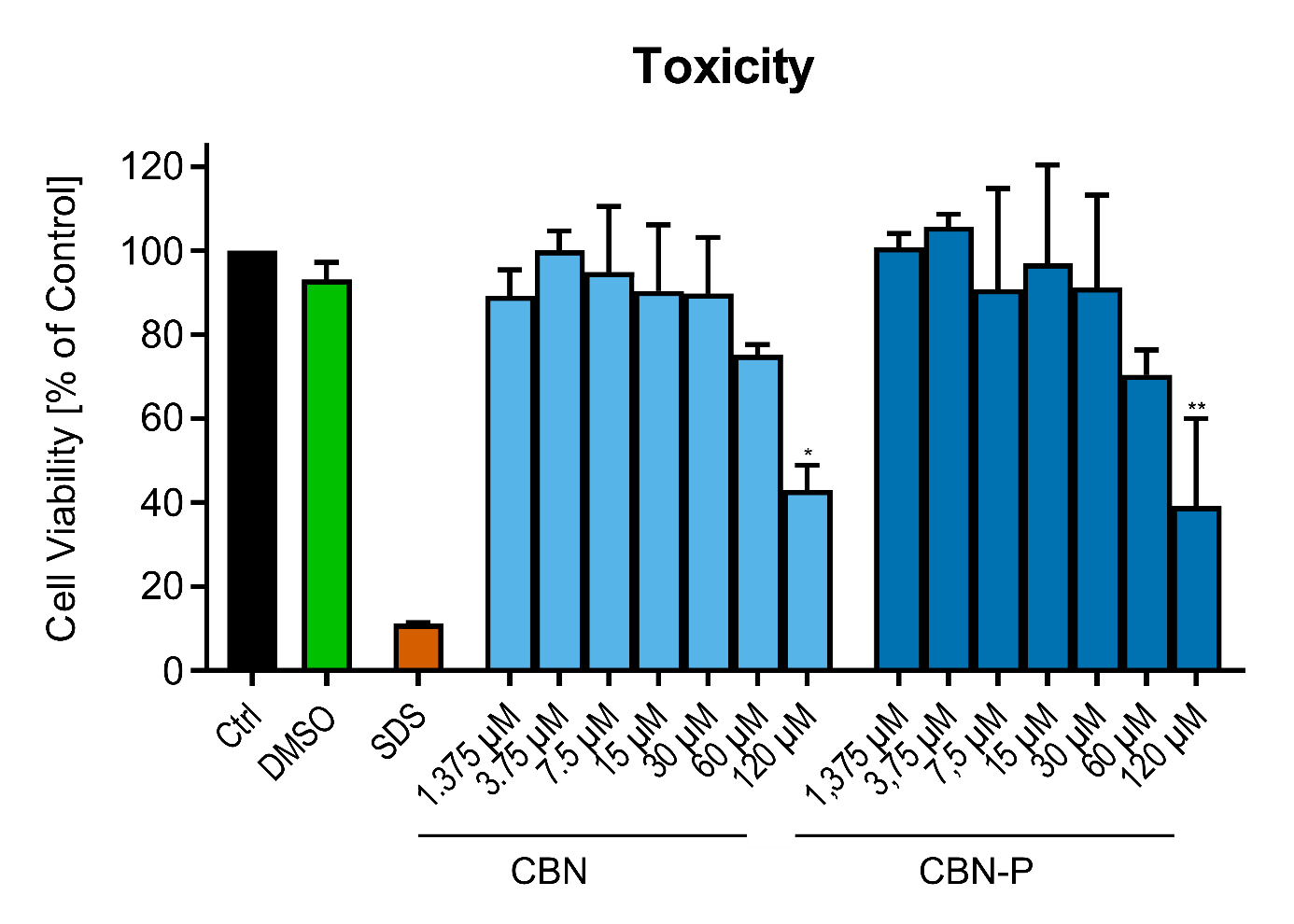


**Figure S2.** Neurotoxicity of CBN and CBN-P after treatment of the HT22 cells for 2 h. Data are presented as means ± SEM of three independent experiments. Statistical analysis was performed using One-way ANOVA followed by Dunnett’s multiple comparison post-test using GraphPad Prism 10 referring to untreated control cells. Levels of significance: **p < 0.05; **p < 0.01; ***p < 0.001, ****p<0.0001*.

# Figure S3: Mitochondrial Calcium Homeostasis


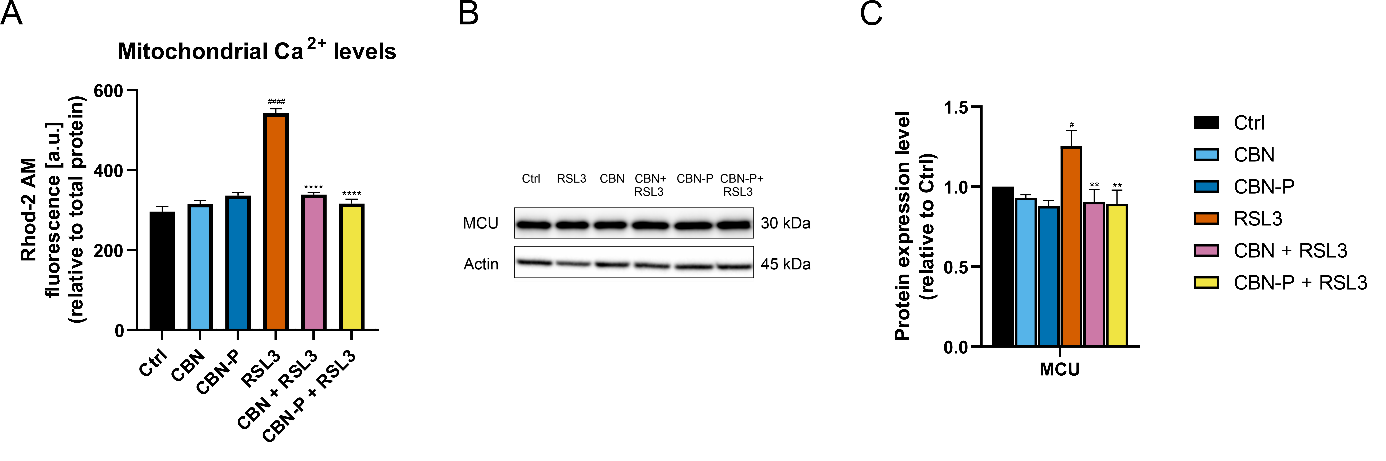


**Figure S3.** CBN and CBN-P prevent Ca^2+^ influx induced by oxytosis/ferroptosis in HT22 cells. (**A**) Mitochondrial Ca^2+^ levels upon different treatment conditions of the cells for 16 h. CBN and CBN-P were used at 5 µM, RSL3 was used at 150 nM. Data were normalized to total protein/well and are the mean of 16 replicates per condition ± SEM. (**B**) Western blot data of MCU and actin after treatment of cells with CBN or CBN-P at 5 µM and RSL3 at 250 nM for 16 h (**C**) Densitometric quantification of the Western blots. Data were normalized to actin and are the mean ± SEM. All data were analyzed by one-way ANOVA with Dunnett’s multiple comparison test. #p < 0.05, ##p < 0.01, ###p < 0.001, ####p < 0.0001 relative to vehicle control; *p < 0.05, **p < 0.01, ***p < 0.001, ****p < 0.0001 relative to the RSL3 treatment.

# Figure S4: Mitochondrial Biogenesis


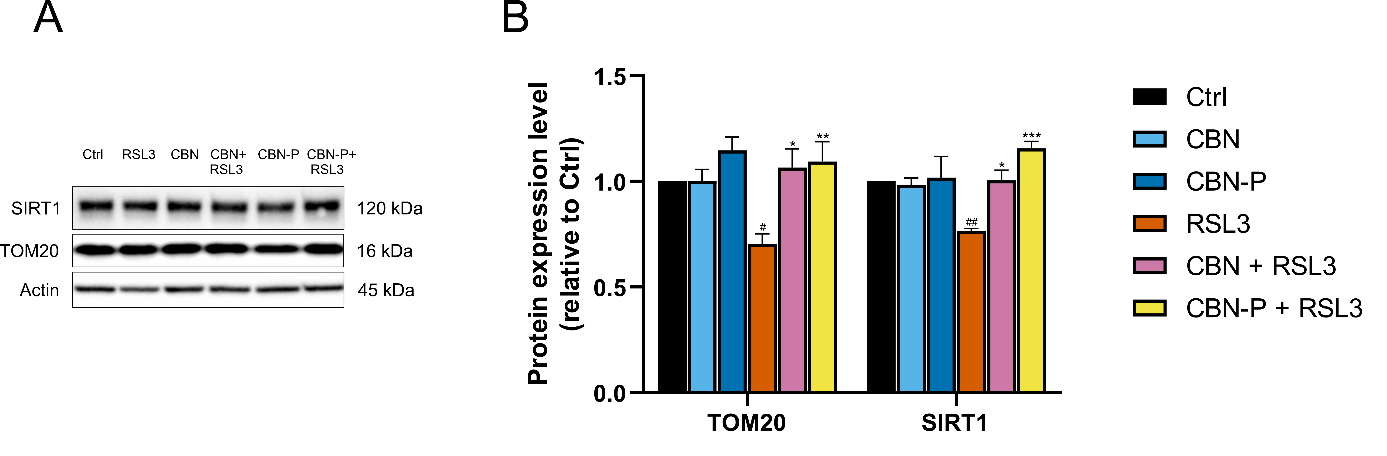


**Figure S4.** (**A**) Western blot data of SIRT1, TOM20, and actin after treatment of cells with CBN or CBN-P at 5 µM and RSL3 at 250 nM for 16 h. (**B**) Densitometric quantification of the Western blots. Data were normalized to actin and are the mean ± SEM. All data were analyzed by one-way ANOVA with Dunnett’s multiple comparison test. #p < 0.05, ##p < 0.01, ###p < 0.001, ####p < 0.0001 relative to vehicle control; *p < 0.05, **p < 0.01, ***p < 0.001, ****p < 0.0001 relative to the RSL3 treatment.

# Figure S5: Mitochondrial Dynamics


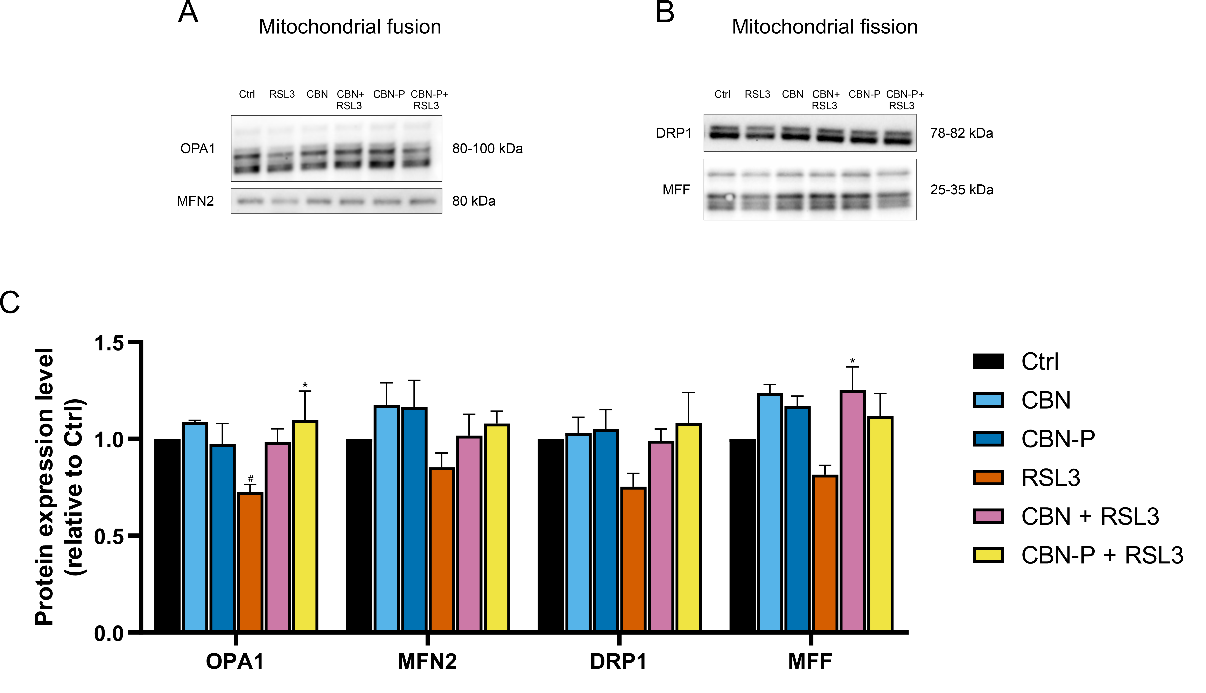


**Figure S5**. (A, B) Western blot data of OPA1, MFN2, DRP1, and MFF (n = 4). Protein levels were measured upon different treatment conditions of the cells for 16 h. (C) Densitometric quantification of the Western blots. Data were normalized to total protein and are the mean ± SEM. Data were analyzed by one-way ANOVA with Tukey’s multiple comparison test. *#p<0.05* relative to vehicle control; **p < 0.05* relative to the RSL3 treatment.

# Figure S6: Click-CLEM Image of DMSO Control


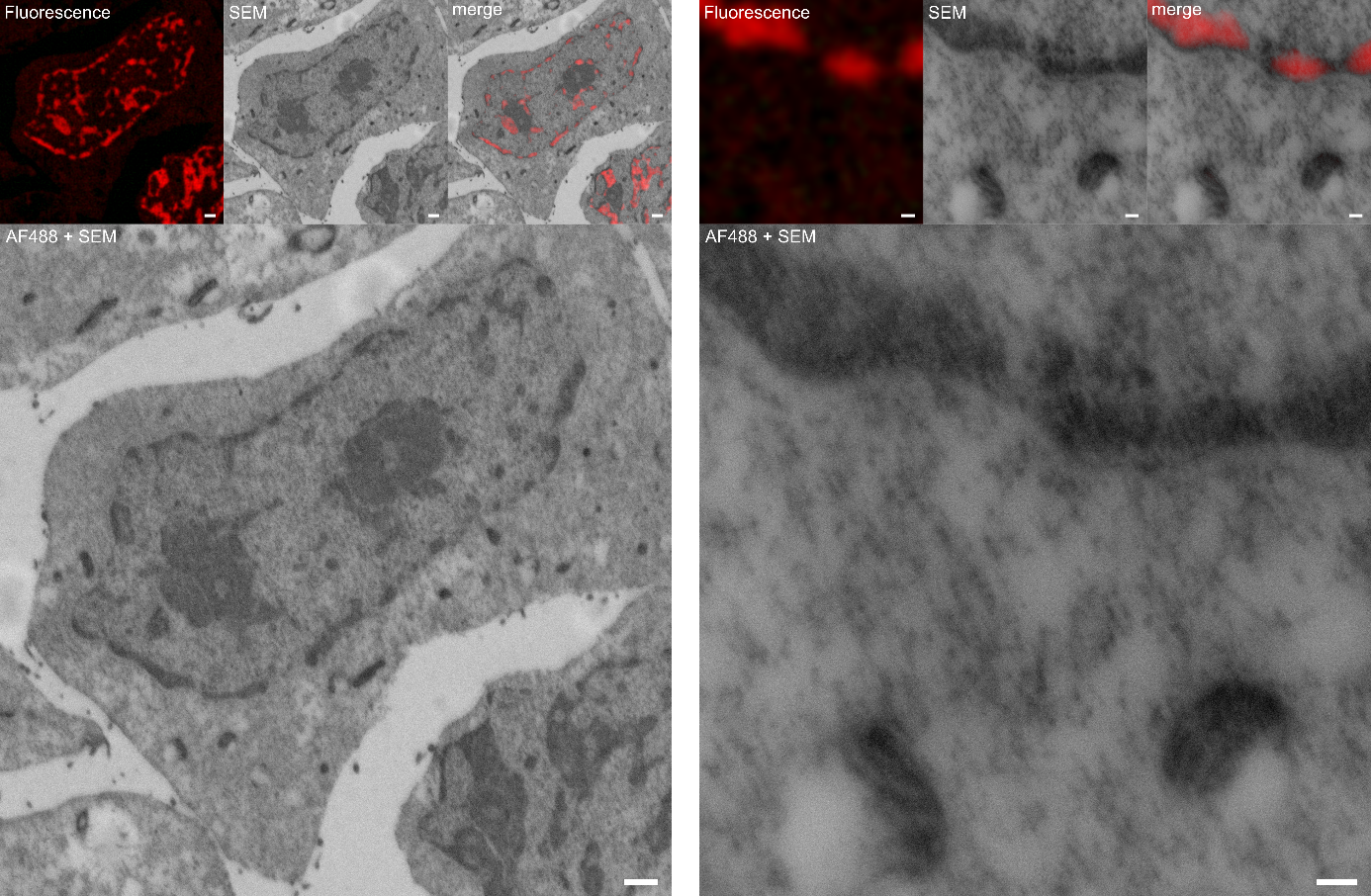


**Figure S6.** Click-correlative light and electron microscopy images of HT22 cells treated with DMSO. Green signal: AF488 attached to the probe; red signal: methyl green (DNA). Upper row shows the image of the merged fluorescence channels after channel alignment taken with SIM, the SEM image, and the merged image showing both fluorescence channels correlated with the SEM image after unbiased correlation; AF488+SEM only displays the probe signal and the SEM image. Images on the right are higher magnified sections of images on the left. Scale bar equals 1 µm (left) or 100 nm (right).

# Figure S7: Western blot analysis for CuAAC control of CBN-P


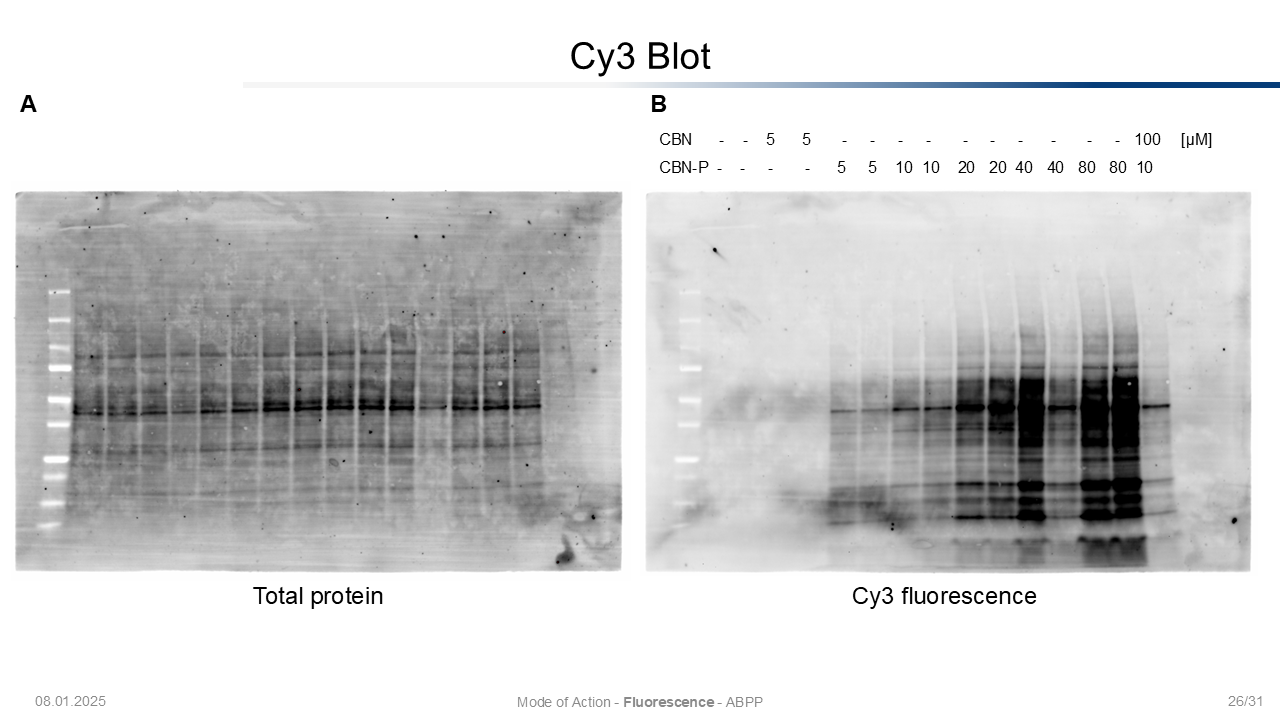


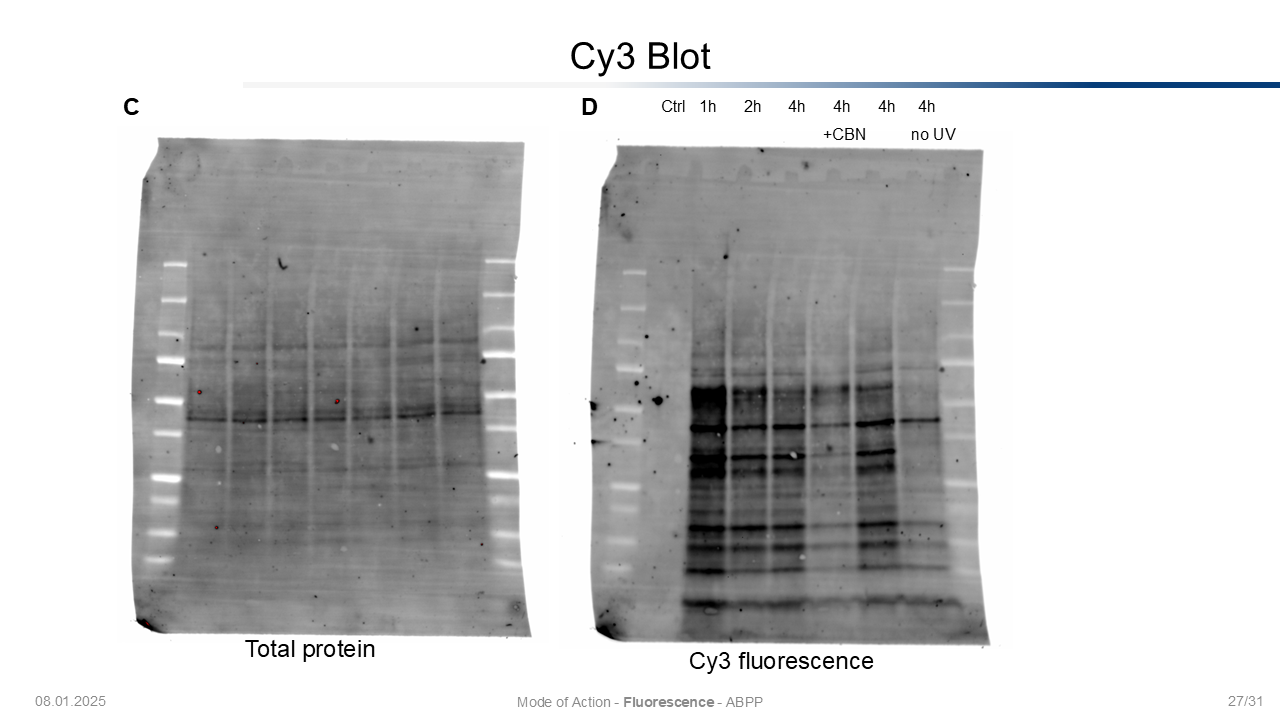


**Figure S7.** Western blots of cells incubated with DMSO or CBN-P, with or without pre-treatment with CBN and subsequent CuAAC with Cy3. (**A**) Total protein image of the membrane in (**B**), lane 12 was deficient. (**B**) Fluorescence signal of Cy3 shows increasing intensity with increasing CBN-P concentration. (**C**) Total protein of membrane in (**D**) showing equal amounts of protein in each sample. (**D**) Fluorescence signal of Cy3 after treatment with 10 µM CBN-P. Highest number of crosslinked proteins can be seen after 1 h of treatment. Pre-treatment with CBN decreases the fluorescence signal.

# Figure S8: Mass Spectrometric Identification of Target Candidates


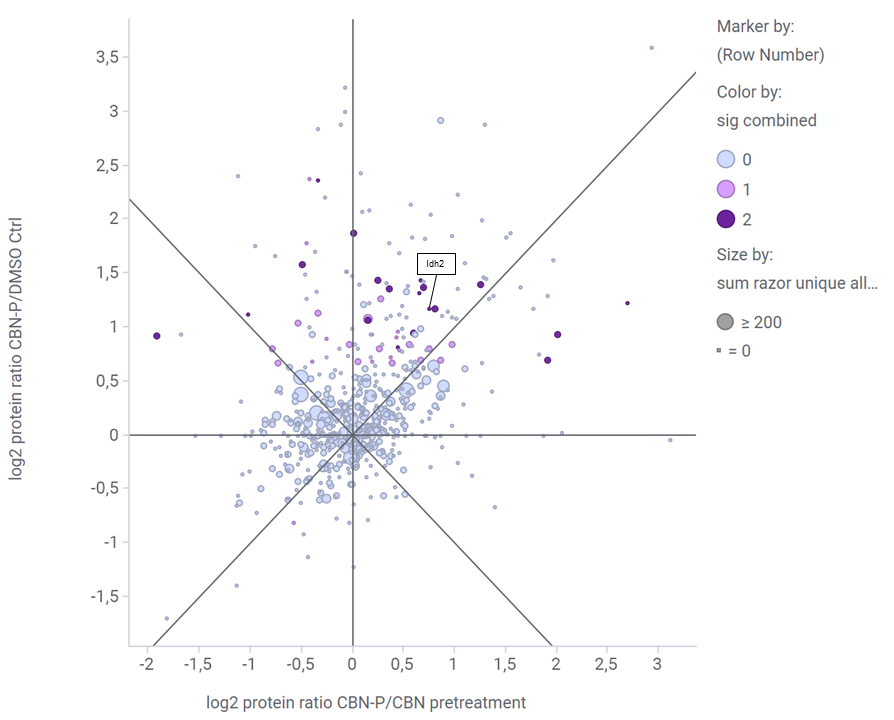


**Figure S8.** Mass spectrometric identification of target candidates. Log2 transformed protein ratios sample/DMSO control and sample/CBN pretreatment with each two replicates are shown. Summed significance values were used to identify the best target candidates. The size of the dots corresponds to the number of razors and unique peptides of a protein.

# Figure S9: Efficacy of IDH2 Knockdown


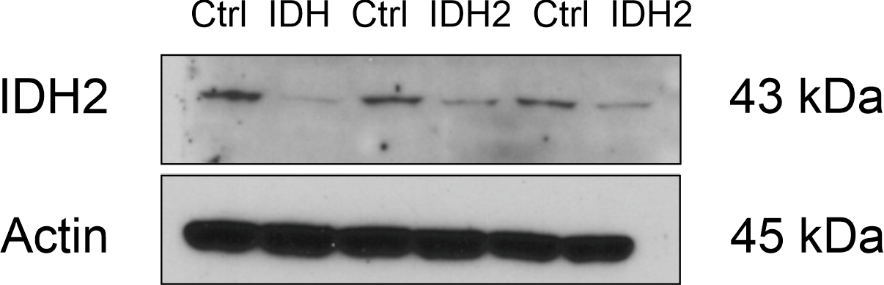


**Figure S9.** Western blot data of HT22 cells after treatment with control siRNA or IDH2 siRNA.

# HPLC-Chromatograms for Purity Control of the Target Compound **CBN-P**


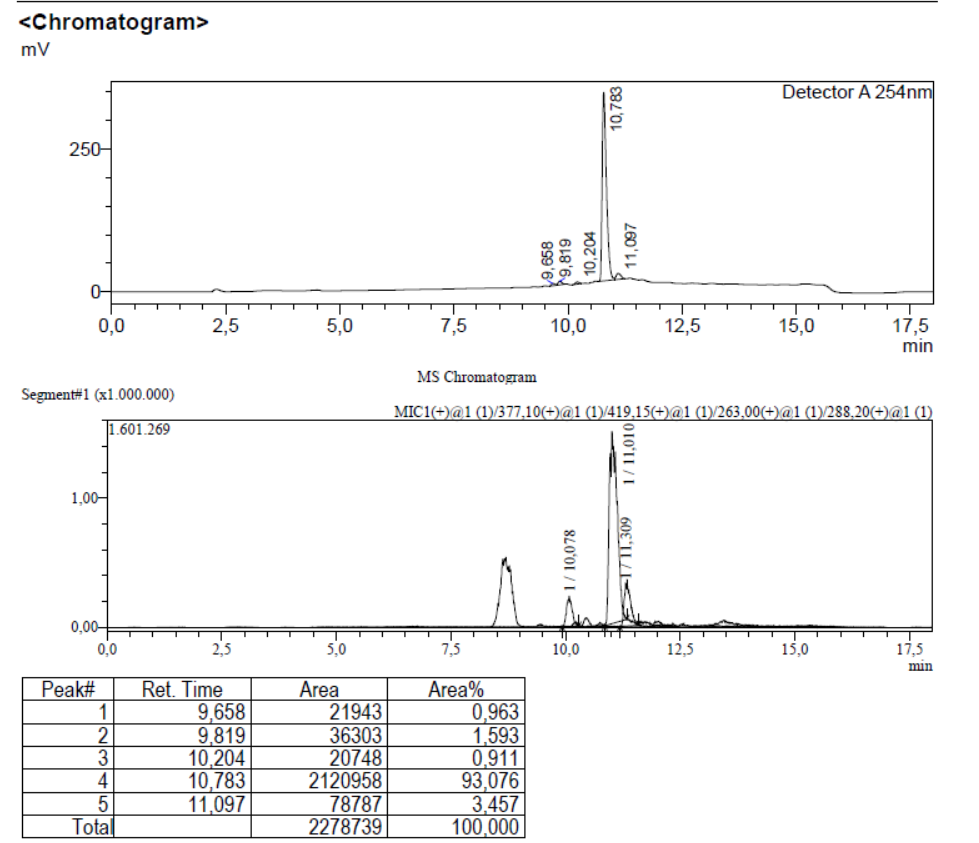


# NMR-Spectra of the Target Compounds **CBN-P**


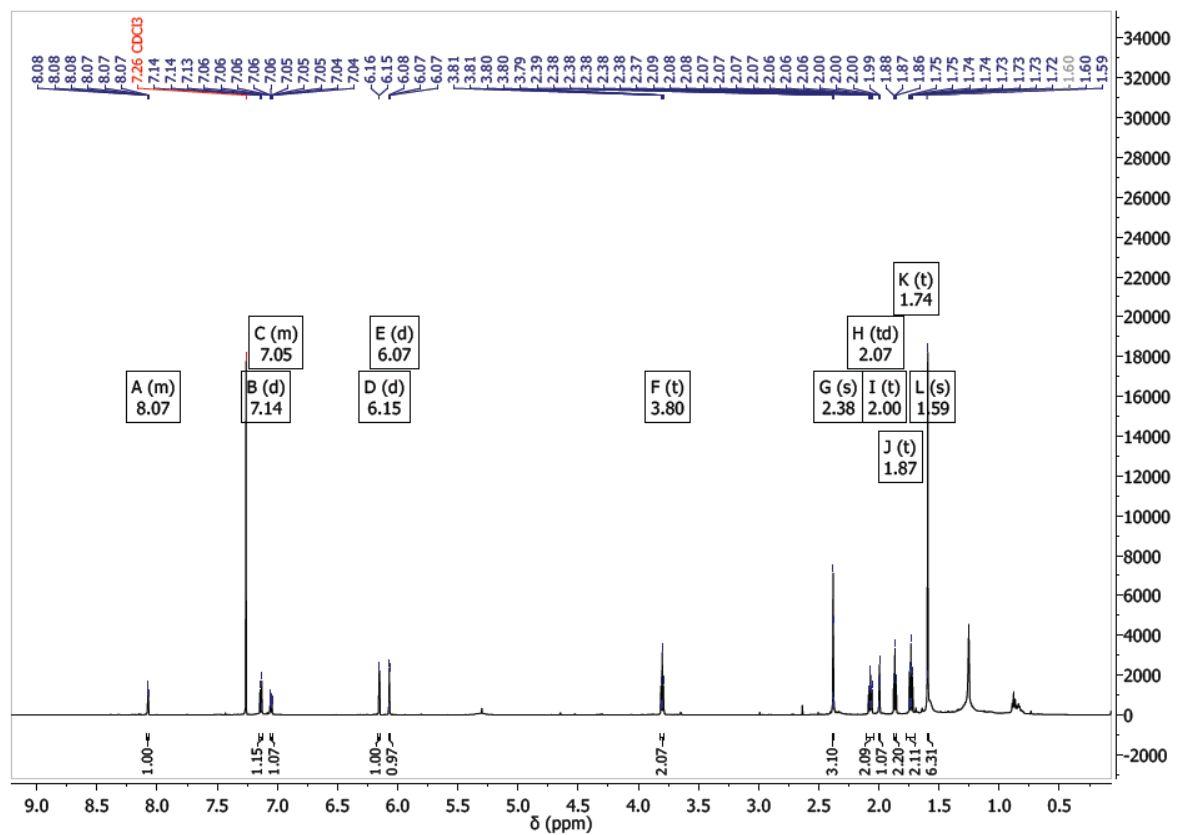

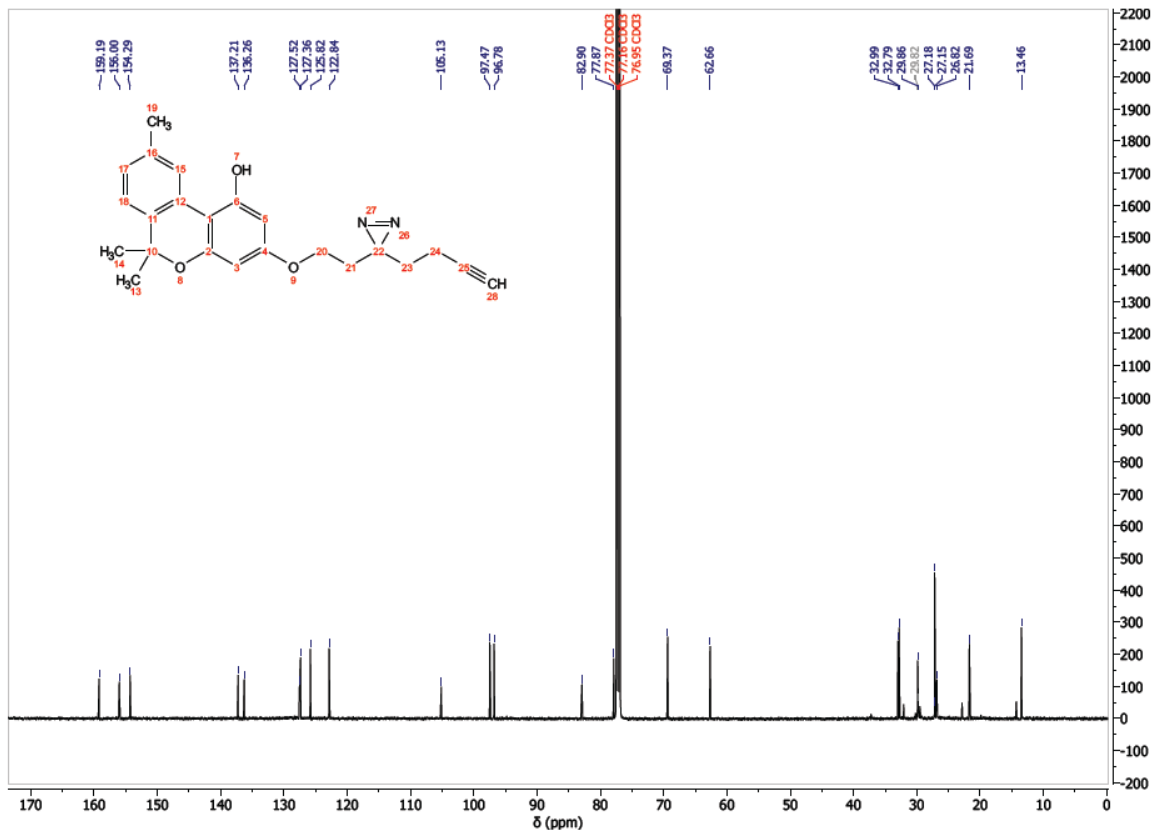


References

[1] D. Caprioglio, D. Mattoteia, A. Minassi, F. Pollastro, A. Lopatriello, E. Muňoz, O. Taglialatela-Scafati, G. Appendino, One-Pot Total Synthesis of Cannabinol via Iodine-Mediated Deconstructive Annulation, Org. Lett. 21 (2019) 6122–6125. https://doi.org/10.1021/acs.orglett.9b02258.

[2] L. Huang, D.B. McClatchy, P. Maher, Z. Liang, J.K. Diedrich, D. Soriano-Castell, J. Goldberg, M. Shokhirev, J.R. Yates, D. Schubert, A. Currais, Intracellular amyloid toxicity induces oxytosis/ferroptosis regulated cell death, Cell Death Dis. 11 (2020) 828. https://doi.org/10.1038/s41419-020-03020-9.

[3] S.M. Markert, V. Bauer, T.S. Muenz, N.G. Jones, F. Helmprobst, S. Britz, M. Sauer, W. Rössler, M. Engstler, C. Stigloher, 3D subcellular localization with superresolution array tomography on ultrathin sections of various species, Methods Cell Biol. 140 (2017) 21–47. https://doi.org/10.1016/bs.mcb.2017.03.004.

[4] D. Prieto, G. Aparicio, P.E. Morande, F.R. Zolessi, A fast, low cost, and highly efficient fluorescent DNA labeling method using methyl green, Histochem. Cell Biol. 142 (2014) 335–345. https://doi.org/10.1007/s00418-014-1215-0.

[5] E.S. Reynolds, The use of lead citrate at high pH as an electron-opaque stain in electron microscopy, J. Cell. Biol. 17 (1963) 208–212. https://doi.org/10.1083/jcb.17.1.208.

[6] C.S. Hughes, S. Moggridge, T. Müller, P.H. Sorensen, G.B. Morin, J. Krijgsveld, Single-pot, solid-phase-enhanced sample preparation for proteomics experiments, Nat. Protoc. 14 (2019) 68–85. https://doi.org/10.1038/s41596-018-0082-x.

[7] J. Rappsilber, Y. Ishihama, M. Mann, Stop and go extraction tips for matrix-assisted laser desorption/ionization, nanoelectrospray, and LC/MS sample pretreatment in proteomics, Anal. Chem. 75 (2003) 663–670. https://doi.org/10.1021/ac026117i.

[8] J. Cox, M. Mann, MaxQuant enables high peptide identification rates, individualized p.p.b.-range mass accuracies and proteome-wide protein quantification, Nat. Biotechnol. 26 (2008) 1367–1372. https://doi.org/10.1038/nbt.1511.

[9] J. Cox, M.Y. Hein, C.A. Luber, I. Paron, N. Nagaraj, M. Mann, Accurate proteome-wide label-free quantification by delayed normalization and maximal peptide ratio extraction, termed MaxLFQ, Mol. Cell. Proteomics 13 (2014) 2513–2526. https://doi.org/10.1074/mcp.M113.031591.
